# A discrete ordinates Boltzmann solver for application to inverse planning of photons and protons

**DOI:** 10.1088/1361-6560/acf4de

**Published:** 2023-09-13

**Authors:** James L Bedford

**Affiliations:** 1 Joint Department of Physics, The Institute of Cancer Research and The Royal Marsden NHS Foundation Trust, London SM2 5PT, United Kingdom

**Keywords:** inverse planning, discrete ordinates, linear Boltzmann transport equations, proton therapy, VMAT

## Abstract

The aim of this work is to develop a discrete ordinates Boltzmann solver that can be used for calculation of absorbed dose from both photons and protons within an inverse planning optimiser, so as to perform accurate dose calculation throughout the whole of the inverse planning process. With photons, five transport sweeps were performed to obtain scattered photon fluence, and unscattered electron fluence was then obtained and used as a fixed source for solution of the electron transport equations. With protons, continuous slowing down was treated as a fixed source, and five transport sweeps were used to calculate scattered fluence. The total electron or proton fluence was multiplied by the stopping power ratio for the transport medium to obtain absorbed dose. The method was evaluated in homogeneous media and in a lung case where the planning target volume was surrounded by low-density lung material. Photon arc, proton passive scattering and proton arc treatments were considered. The results were compared to a clinically validated convolution dose calculation for photons, and with an analytical method for protons. In water-equivalent media, the discrete ordinates method agrees with the alternative algorithms to within 2%. Convergence is found to be sufficiently complete for water-, lung- and bone-equivalent materials after five iterations. The dose calculated by the relatively simple angular quadrature is seen to be very close to that calculated by a more comprehensive quadrature. For inhomogeneous lung plans, the method shows more heterogeneity of dose to the planning target volume than the comparative methods. The discrete ordinates Boltzmann solver provides a general framework for dose calculation with both photons and protons. The method is suitable for incorporation into an inverse planning optimiser, so that accurate dose calculation in a heterogeneous medium can be obtained throughout inverse planning, with the result that the final dose distribution is as predicted by the optimiser.

## 1. Introduction

In the last decade, the value of the discrete ordinates method of solving the linear Boltzmann transport equations (LBTEs) as a means of absorbed dose calculation in radiotherapy has been widely appreciated (Gifford *et al* 2006, Vassiliev *et al* 2010, Bedford 2019). Compared to Monte Carlo simulation, which is an alternative method of solving the LBTE, the discrete ordinates method is deterministic and therefore provides solutions in which stochastic uncertainty is absent (Vassiliev 2017). The Acuros implementation (Varian Medical Systems, Palo Alto, CA) has also led to the widespread application in many centres, together with numerous comparisons with Monte Carlo simulation (Han *et al* 2011, 2013, Fogliata *et al* 2012, Hoffmann *et al* 2018). The inclusion of a magnetic field in the physical modelling has enabled application to MR-guided radiotherapy (St Aubin *et al* 2015, 2016, Zelyak *et al* 2018a, 2018b).

However, the method has much greater potential for application in radiotherapy than has so far been realised. Two particular impediments to its application can be identified, notably (a) the difficulty of applying it to proton therapy, and (b) the difficulty of its application to inverse planning. In the former, that of application to proton therapy, the difficulty is that proton transport is largely unidirectional, with relatively little energy loss until the Bragg peak, where the particles change direction considerably, and lose all of their remaining energy. Modelling this process using LBTE without any prior assumptions such as continuous slowing down approximation requires many energy and direction ordinates, most of which, for the initial part of the beam trajectory, are irrelevant, and a drain on memory resources (Sanchez and McCormick 2004, Uilkema 2012).

With regard to the latter impediment, that of inverse planning, one of the advantages of the discrete ordinates method is that the absorbed dose due to all control points of all beams can be calculated by one solution of the transport equations. This is efficient for volumetric modulated arc therapy (VMAT), where there are many control points, but a difficulty arises with inverse planning, where the dose distribution due to each fluence element, or bixel, of each beam is invariably required for the inverse planning algorithm. This can be accomplished by the discrete ordinates method by repeated applications, but is very inefficient.

The ultimate aim of the project described in this paper is to address these issues so as to provide a discrete ordinates solver which can be used for proton therapy and also applied efficiently to inverse planning. For proton therapy, the approach is to treat the initial highly directional proton trajectory as a fixed source based on semi-empirical methods, mostly reserving the discrete ordinates solution for the Bragg peak. With inverse planning, the approach is to use the discrete ordinates method to perform regular calculations of the dose due to the entire plan, thereby utilising the efficiency of the method, and then to use a convolution method to provide the bixel-based doses required for inverse planning purposes. Compatibility of the LBTE solver with the convolution method is therefore required. For all of these methods, a short computation time and a reasonable memory allocation are needed, and it is therefore necessary to make some simplifications to produce a practical solution. The main simplification, compared to the Acuros implementation (Vassiliev *et al* 2010), is to use fewer directional and energy ordinates in the quadrature.

As this project is substantial, it is not feasible to present all of the work in one paper. This paper therefore concentrates on the methods and validation of Boltzmann solutions with both photon and proton beams. Actual incorporation of the methods into inverse planning is not described, although this goal is in the background throughout. The form of the paper is consequently as follows: the Boltzmann solver is described firstly for photon beams and then for proton beams. The adequacy of the iterative scheme and the quadrature is demonstrated for homogeneous media. The method is then applied to a lung case, where the tissue inhomogeneity is particularly pronounced. In this case, photon arcs (VMAT), fixed (passively scattered) proton beams and proton arcs are used. The results when using the Boltzmann approach are compared with those produced by alternative algorithms already implemented in the inverse planning context.

## 2. Materials and methods

### 2.1. Head model

The LBTE solver proceeded by projecting unscattered photons or protons into the patient model. In the case of photons, the fluence was calculated using a dual-source model of a Versa HD linear accelerator (Elekta AB, Stockholm) with a source-axis distance of 1000 mm (Bedford *et al* 2013). A 6 MV flattened beam was considered in this work, although the approach can also be used for a flattening filter-free beam. For each of the two radiation sources in the dual-source model, representing primary photons and head scatter, divergent rays were traced throughout the patient model according to a regular Cartesian grid defined at the isocentric plane (Siddon 1985, Bedford *et al* 2022). The intensities of the rays were determined from the off-axis position of the rays according to a lookup table defined in the beam data for the dose calculation model (Bedford 2002). A fluence grid at the position of the collimating device was defined for each source in the model and the contributions of the rays to the elements of the fluence map were determined, taking into account the divergence of the rays. The resulting fluence was then convolved with a Gaussian source function of specified intensity and width, again determined as part of the beam data. For the Versa HD accelerator, the primary source was modelled as having a width (standard deviation) of 0.8 mm and a length of 1.0 mm, while the secondary source, at 150 mm from the primary source, was modelled as having a width and length of 18.0 mm. Further details of the source model are given elsewhere (Bedford *et al* 2022).

In the case of passively scattered protons, the same source model was used, but with parameters representing the 230 MeV beam of a double-scattering system (Ion Beam Applications S.A., Louvain-la-Neuve, Belgium) (Slopsema 2012), with a single source having a source-axis distance of 2300 mm. The width and length of the source were both modelled as 30.0 mm (Slopsema *et al* 2014). For proton arcs, the pencil beam width (two standard deviations) was taken as 10.0 mm.

### 2.2. Quadrature

The angular quadrature for discrete ordinates was based upon the IEC61217 convention for gantry and couch angles, so that the different discrete directions could be conveniently addressed using the corresponding beam orientations. The ‘gantry angles’ of the quadrature were 0.0°, 47.5°, 90.0°, 132.5°, 180.0°, 227.5°, 270.0° and 312.5°, i.e. approximately 45° spacing, with a bias of the diagonal directions of 2.5° towards the lateral directions. The ‘couch angles’ were spaced at 45° from 270° to 45° through 0°. The couch angles, *c*, and gantry angles, *g*, were considered as a spherical coordinate system, with each combination of *c* and *g* giving a direction vector, Ω:

\begin{eqnarray*}{\boldsymbol{\Omega }}={\left[\begin{array}{cc}c &amp; g\end{array}\right]}^{T}.\end{eqnarray*}

**Figure 1.**
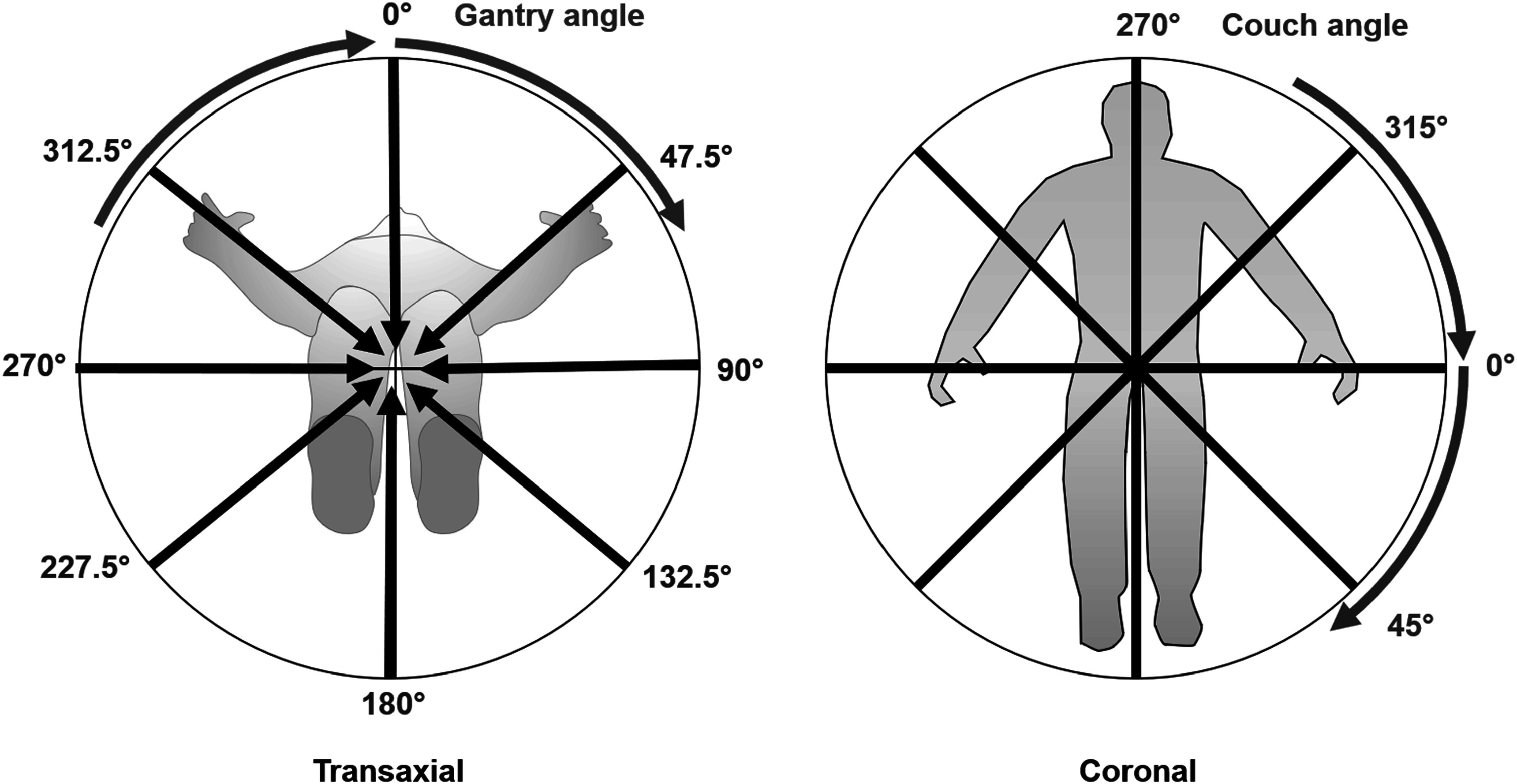
Illustration of the angular quadrature used in this work. The set of directions shown in the transaxial view is repeated at the four angles in the coronal view, thereby covering the 4*π* steradians of direction space.

This range of gantry and couch angles permitted the complete orientation space to be addressed, corresponding to the arrangement of lines of latitude and longitude around the globe (figure 1). This was useful for visualisation purposes, but had the drawback that the solid angle occupied by each orientation varied between the ordinates, reducing towards the orientations with gantry 0° and 180°. At these two orientations, the variation of the couch angles led to multiple ordinates at identical orientation. The couch angles of the ordinates at gantry 0° and 180° were therefore constrained to 0°. A simple uniform weighting was used for the angular quadrature, the small bias of the diagonal gantry angles towards 90° or 270° being used to ensure even coverage of the orientation space. For an ordinate with couch angle *c* and gantry angle *g*, the weighting was given by:

\begin{eqnarray*}\begin{array}{c}{w}_{cg}=0,\,\left(g=0,180\right)\cap c\ne 0\\ =\displaystyle \frac{4\pi }{CG},\,\mathrm{otherwise},\end{array}\end{eqnarray*}

where *C* (=4) and *G* (=8) represented the total number of couch and gantry angles in the quadrature. For Compton scattering of photons, an additional weighting factor was used, which is described in section 2.3. Note that the angular quadrature was fixed in space and used for all segments of the VMAT arcs, so that a single application of the LBTE could be used to calculate dose for the complete plan, with maximum efficiency.

Energy was discretised into *G* groups (*g* = 1, 2, 3, …, *G*) using the multigroup method (Lewis and Miller 1984). Each group was characterised by a discrete energy, *E*_*g*_. For photons, the energy quadrature was arranged such that *E*_*g*_ increased in 0.5 MeV steps up to 4.0 MeV, followed by 1.0 MeV steps up to 10.0 MeV. For protons, the energy quadrature was arranged in 5 MeV steps from 5 to 250 MeV. The uppermost energies for both photons and protons were redundant, but were included for generality, should higher energy particles need to be considered. The group boundaries lay midway between the discrete energies, *E*_*g*_, with the upper boundary for group g being ${E}_{g}^{+}$ and the lower energy boundary being ${E}_{g}^{-}.$ As in previous work (Gifford *et al* 2006), the continuous fluence ${\mathrm{\Phi }}\left({\bf{r}},E,{\boldsymbol{\Omega }}\right),$ where **Ω** referred to the angular ordinate, was divided into a fluence component and an energy component:

\begin{eqnarray*}{\mathrm{\Phi }}\left({\bf{r}},E,{\boldsymbol{\Omega }}\right)={{\mathrm{\Phi }}}_{g}\left({\bf{r}},{\boldsymbol{\Omega }}\right)f\left(E\right),\end{eqnarray*}

with $f\left(E\right)$ then being selected to be constant within each energy range:

\begin{eqnarray*}f\left(E\right)=1/\left({E}_{g}^{+}-{E}_{g}^{-}\right).\end{eqnarray*}

Note that a consequence of this was that the total fluence for group *g*, as given by the integral $\displaystyle {\int }_{{E}_{g}^{-}}^{{E}_{g}^{+}}{\mathrm{\Phi }}\left({\bf{r}},E,{\boldsymbol{\Omega }}\right)dE,$ was simply equal to ${{\mathrm{\Phi }}}_{g}\left({\bf{r}},{\boldsymbol{\Omega }}\right),$ which simplified the computational handling of the energy quadrature.

### 2.3. Photon transport

The differences between transport of photons and protons in the LBTE solver were mostly found in the initial transport of unscattered fluence, and they are therefore outlined separately. With regard to photons, for each ray cast from the head model, equivalent path length was determined, from which an exponential decay was calculated. The energy for this was calculated from an energy spectrum and the attenuation coefficients were taken from ICRU46 (International Commission on Radiation Units and Measurements 1992). Taking the position in the beam’s eye view to be represented by coordinates *x* and *y*, and the depth in the patient to be represented by *z*, the unscattered fluence, ${{\mathrm{\Phi }}}_{\gamma }\left({\bf{r}},{E}_{\gamma }\right),$ at position ${\bf{r}}\left(x,y,z\right),$ with quadrature energy ${E}_{\gamma }$ was calculated as:

\begin{eqnarray*}{{\mathrm{\Phi }}}_{\gamma }\left({\bf{r}},{E}_{\gamma }\right)=\displaystyle \frac{1}{{z}^{2}}\left({\omega }\left(x,y\right)\otimes s\left(x,y\right)\right){\psi }\left(x,y\right){\eta }\left({E}_{\gamma }\right)\exp \left(-{{\mu }}_{0}\left({E}_{\gamma }\right)z-{\mu }{^{\prime} }\sqrt{{x}^{2}+{y}^{2}}z\right),\end{eqnarray*}

where ${\omega }\left(x,y\right)$ was a two-dimensional step function representing the beam aperture, $s\left(x,y\right)$ was the Gaussian source profile, ${\psi }\left(x,y\right)$ was the tabulated fluence profile of the radiation exiting the accelerator head, ${\eta }\left({E}_{\gamma }\right)$ was the energy spectrum, ${{\mu }}_{0}\left({E}_{\gamma }\right)$ was the attenuation coefficient and ${\mu }{^{\prime} }$ represented the off-axis softening of the beam. Note that *ω* and ${\mu }{^{\prime} }$ were actually functions of beam aperture size and shape, but that dependency is suppressed in the equation for simplicity.

To assign the unscattered photon fluence to the quadrature orientations, the fluence calculated by equation (5) was assigned to the four orientation ordinates encompassing the beam orientation in a process analogous to bilinear interpolation. If the gantry angles of the ordinates were *g*_1_ and *g*_2_, the couch angles were *c*_1_ and *c*_2_, and the gantry and couch angles of the beam were *g* and *c* respectively, the fluence assigned to each of the four ordinates was given by:

\begin{eqnarray*}{{\mathrm{\Phi }}}_{11}=\displaystyle \frac{{g}_{2}-g}{{g}_{2}-{g}_{1}}.\displaystyle \frac{{c}_{2}-c}{{c}_{2}-{c}_{1}},\end{eqnarray*}

\begin{eqnarray*}{{\mathrm{\Phi }}}_{21}=\displaystyle \frac{g-{g}_{1}}{{g}_{2}-{g}_{1}}.\displaystyle \frac{{c}_{2}-c}{{c}_{2}-{c}_{1}},\end{eqnarray*}

\begin{eqnarray*}{{\mathrm{\Phi }}}_{12}=\displaystyle \frac{{g}_{2}-g}{{g}_{2}-{g}_{1}}.\displaystyle \frac{c-{c}_{1}}{{c}_{2}-{c}_{1}},\end{eqnarray*}

\begin{eqnarray*}{{\mathrm{\Phi }}}_{22}=\displaystyle \frac{g-{g}_{1}}{{g}_{2}-{g}_{1}}.\displaystyle \frac{c-{c}_{1}}{{c}_{2}-{c}_{1}},\end{eqnarray*}

where the subscripts on Φ referred to the gantry and couch ordinates respectively.

The photon fluence was initially set equal to the unscattered fluence as calculated from equation (5) and (6). The dose calculation then solved the LBTEs for photons, to give the scattered fluence. Defining ${{\boldsymbol{\Omega }}}_{\gamma }$ to be a unit normal in the direction of interest, **r** to be the position of interest and ${E}_{\gamma }$ to be the photon energy of interest:

\begin{eqnarray*}\begin{array}{c}{{\boldsymbol{\Omega }}}_{\gamma }\cdot {\mathrm{\nabla }}{{\mathrm{\Phi }}}_{\gamma }^{\mathrm{scat}}\left({\bf{r}},{{\boldsymbol{\Omega }}}_{\gamma },{E}_{\gamma }\right)\\ =\,{{\rho }}_{e}\left({\bf{r}}\right)\displaystyle {\int }_{0}^{\infty }\displaystyle {\int }_{4\pi }{\tilde{{\sigma }}}_{C,\gamma }\left({E^{\prime} }_{\gamma },{E}_{\gamma },{{\boldsymbol{\Omega }}{\boldsymbol{^{\prime} }}}_{\gamma }\cdot {{\boldsymbol{\Omega }}}_{\gamma }\right){{\mathrm{\Phi }}}_{\gamma }^{\mathrm{scat}}\left({\bf{r}},{{\boldsymbol{\Omega }}{\boldsymbol{^{\prime} }}}_{\gamma },{E^{\prime} }_{\gamma }\right)d{{\boldsymbol{\Omega }}{\boldsymbol{^{\prime} }}}_{\gamma }d{E^{\prime} }_{\gamma }\\ \,-{{\rho }}_{e}\left({\bf{r}}\right){{\sigma }}_{C,\gamma }^{\mathrm{tot}}\left({E}_{\gamma }\right){{\mathrm{\Phi }}}_{\gamma }^{\mathrm{scat}}\left({\bf{r}},{{\boldsymbol{\Omega }}}_{\gamma },{E}_{\gamma }\right)\end{array}\end{eqnarray*}

where ${\rho }_{e}\left({\bf{r}}\right)$ was the electron density at position **r**, ${{\mathrm{\Phi }}}_{\gamma }^{\mathrm{scat}}\left({\bf{r}},{{\boldsymbol{\Omega }}}_{\gamma },{E}_{\gamma }\right)$ was the scattered photon fluence at position **r**, with direction ${{\boldsymbol{\Omega }}}_{\gamma }$ and energy ${E}_{\gamma },$ ${\tilde{\sigma }}_{C,\gamma }\left({E^{\prime} }_{\gamma },{E}_{\gamma },{{\boldsymbol{\Omega }}{\boldsymbol{^{\prime} }}}_{\gamma }\cdot {{\boldsymbol{\Omega }}}_{\gamma }\right)$ was the differential Compton scattering cross section of a photon travelling initially with energy ${E^{\prime} }_{\gamma }$ in direction ${{\boldsymbol{\Omega }}{\boldsymbol{^{\prime} }}}_{\gamma }$ and finally with energy ${E}_{\gamma }$ and direction ${{\boldsymbol{\Omega }}}_{\gamma },$ and ${{\sigma }}_{C,\gamma }^{\mathrm{tot}}\left({E}_{\gamma }\right)$ was the total Compton scattering cross section (Hensel *et al* 2006). The integral term of equation (7) referred to scattered sources and was denoted as ${Q}_{nijk}^{\mathrm{scat}}\left(x,y,z\right),$ where *n* indexed the discrete ordinates in the quadrature and *i*, *j* and *k* indexed the voxels in the patient model:

\begin{eqnarray*}{Q}_{nijk}^{\mathrm{scat}}\left(x,y,z\right)={{\rho }}_{e}\left({\bf{r}}\right)\displaystyle {\int }_{0}^{\infty }\displaystyle {\int }_{4\pi }{\tilde{{\sigma }}}_{C,\gamma }\left({E^{\prime} }_{\gamma },{E}_{\gamma },{{\boldsymbol{\Omega }}{\boldsymbol{^{\prime} }}}_{\gamma }\cdot {{\boldsymbol{\Omega }}}_{\gamma }\right){{\mathrm{\Phi }}}_{\gamma }^{\mathrm{scat}}\left({\bf{r}},{{\boldsymbol{\Omega }}{\boldsymbol{^{\prime} }}}_{\gamma },{E^{\prime} }_{\gamma }\right)d{{\boldsymbol{\Omega }}{\boldsymbol{^{\prime} }}}_{\gamma }d{E^{\prime} }_{\gamma }.\end{eqnarray*}

In practice, the integrals of equation (7) were discrete sums over the discrete ordinates. Moreover, a particle undergoing a scattering event for the particular angle between one direction of transport and another, with cosine ${\boldsymbol{\Omega }}{\boldsymbol{^{\prime} }}\cdot {\boldsymbol{\Omega }},$ and ending with energy *E*, had a specific initial energy, $E^{\prime} ,$ as governed by the kinematics of the scattering event. Consequently, the double integral collapsed to a single summation:

\begin{eqnarray*}{Q}_{nijk}^{\mathrm{scat}}\left(x,y,z\right)\cong {\rho }_{e}\left({\bf{r}}\right)\displaystyle \sum _{n=1}^{N}{\tilde{{\sigma }}}_{C,\gamma }\left({E^{\prime} }_{\gamma },{E}_{\gamma },{{\boldsymbol{\Omega }}{\boldsymbol{^{\prime} }}}_{\gamma }\cdot {{\boldsymbol{\Omega }}}_{\gamma }\right){{\mathrm{\Phi }}}_{\gamma }^{\mathrm{scat}}\left({\bf{r}},{{\boldsymbol{\Omega }}{\boldsymbol{^{\prime} }}}_{\gamma },{E^{\prime} }_{\gamma }\right).\end{eqnarray*}

This required the initial interaction energy to be calculated for a given transition between the *N* angular ordinates and a given final energy. The equations given by Hensel *et al* (2006) were largely used for this purpose, leading to an *N* × *N* matrix of initial energies.

The differential and total scattering cross sections were also precalculated and stored at the beginning of each photon calculation. Again, the differential scattering cross sections were only needed for the specific angles between each of the ordinate directions, *N* × *N* items.

The differential cross sections for Compton scattering of both photons and electrons, together with the total cross section, were taken from Hensel *et al* (2006), based on Davisson and Evans (1952), and were straightforward to handle. Due to the coarse sampling of the angular quadrature, it was necessary to adjust the relative contributions of forward-scattered and large angle-scattered photons. Defining the cosine of the photon scattering angle to be *θ*, a threshold, *θ*_0_, was set. Then for $\theta \geqslant {\theta }_{0},$ the differential scattering cross sections were weighted by a factor ${f}_{C,\gamma }^{0}$ = 2.0, for $\theta < {\theta }_{0}$ the differential scattering cross sections were weighted by a factor of ${f}_{C,\gamma }$= 10.0 and the total scattering cross section was correspondingly multiplied by a factor of ${f}_{C,\gamma }^{\mathrm{tot}}$ = 3.0. The value of *θ*_0_ was 1.0 in this instance, although lower values were also used in the validation (section 2.10). These factors were used for all types of tissue. The factors mostly affected the gradient and curvature of the depth dose curves, particularly in the region between 50 and 150 mm depth, where photon scatter formed a prominent part. They were chosen by varying them systematically to give good agreement of the calculated dose with measured depth-dose curves. Although this step was not physically exact, the empirical approach yielded satisfactory results.

The equation (7) was solved using a series of transport sweeps (Lewis and Miller 1984). Within each iteration, a transport sweep was carried out for descending energy for each of the discrete ordinates. The sweep proceeded in either the *x*-, *y*- or *z*- direction, depending upon which octant the discrete ordinate direction was located in. If the voxels were indexed by *i*, *j*, *k,* bounded by *x*_1/2_, *x*_3/2_, *…x*_*I*+1/2_, *y*_1/2_, *y*_3/2_, *…y*_*J*+1/2_, *z*_1/2_, *z*_3/2_, *…z*_*K*+1/2_, with dimensions:

\begin{eqnarray*}{\mathrm{\Delta }}{x}_{i}={x}_{i+1/2}-{x}_{i-1/2},\,{\mathrm{\Delta }}{y}_{j}={y}_{j+1/2}-{y}_{j-1/2},\,{\mathrm{\Delta }}{z}_{k}={z}_{k+1/2}-{z}_{k-1/2},\end{eqnarray*}

integrating the transport equation over voxels and dividing by ${\mathrm{\Delta }}{x}_{i}{\mathrm{\Delta }}{y}_{j}{\mathrm{\Delta }}{z}_{k},$ gave the relationship:

\begin{eqnarray*}\begin{array}{c}\displaystyle \frac{{\mu }_{n}}{{\mathrm{\Delta }}{x}_{i}}\left({{\mathrm{\Phi }}}_{n,i+1/2,jk}^{\mathrm{scat}}-{{\mathrm{\Phi }}}_{n,i-1/2,jk}^{\mathrm{scat}}\right)+\displaystyle \frac{{\eta }_{n}}{{\mathrm{\Delta }}{y}_{j}}\left({{\mathrm{\Phi }}}_{ni,j+1/2,k}^{\mathrm{scat}}-{{\mathrm{\Phi }}}_{ni,j-1/2,k}^{\mathrm{scat}}\right)+\displaystyle \frac{{\xi }_{n}}{{\mathrm{\Delta }}{z}_{k}}\left({{\mathrm{\Phi }}}_{nij,k+1/2}^{\mathrm{scat}}-{{\mathrm{\Phi }}}_{nij,k-1/2}^{\mathrm{scat}}\right)\\ +{{\rho }}_{e}\left(x,y,z\right){{\sigma }}_{C,\gamma }^{\mathrm{tot}}{{\mathrm{\Phi }}}_{nijk}^{\mathrm{scat}}\left(x,y,z\right)={Q}_{nijk}^{\mathrm{scat}}\left(x,y,z\right),\end{array}\end{eqnarray*}

where *μ*_*n*_, *η*_*n*_ and *ξ*_*n*_ were the direction cosines of the discrete ordinates, *n* (Hensel *et al* 2006). The following relationship was used to relate the fluence at the centre of the voxel to that at the edge:

\begin{eqnarray*}{{\mathrm{\Phi }}}_{nijk}^{\mathrm{scat}}={{\mathrm{\Phi }}}_{n,i+1/2,jk}^{\mathrm{scat}},\end{eqnarray*}

\begin{eqnarray*}{{\mathrm{\Phi }}}_{nijk}^{\mathrm{scat}}={{\mathrm{\Phi }}}_{ni,j+1/2,k}^{\mathrm{scat}},\end{eqnarray*}

\begin{eqnarray*}{{\mathrm{\Phi }}}_{nijk}^{\mathrm{scat}}={{\mathrm{\Phi }}}_{nij,k+1/2}^{\mathrm{scat}}.\end{eqnarray*}

Note that this represented a hybrid scheme between the diamond difference relationship (Lewis and Miller 1984) and a step scheme: the fluence changed from the side of the voxel with the lower index $i-1/2,$ $j-1/2,$ $k-1/2$ to the centre of the voxel and then levelled off to the side of the voxel with the higher index $i+1/2,$ $j+1/2,$ $k+1/2.$ The standard transport sweep of Lewis and Miller (1984) was then used to solve for scattered fluence:

\begin{eqnarray*}{{\mathrm{\Phi }}}_{nijk}^{\mathrm{scat}}=\displaystyle \frac{\displaystyle \frac{2{\mu }_{n}}{{\mathrm{\Delta }}{x}_{i}}{{\mathrm{\Phi }}}_{n,i-1/2,jk}^{\mathrm{scat}}+\displaystyle \frac{2{\eta }_{n}}{{\mathrm{\Delta }}{y}_{j}}{{\mathrm{\Phi }}}_{ni,j-1/2,k}^{\mathrm{scat}}+\displaystyle \frac{2{\xi }_{n}}{{\mathrm{\Delta }}{z}_{k}}{{\mathrm{\Phi }}}_{nij,k-1/2}^{\mathrm{scat}}+{Q}_{nijk}^{\mathrm{scat}}}{\displaystyle \frac{2{\mu }_{n}}{{\mathrm{\Delta }}{x}_{i}}+\displaystyle \frac{2{\eta }_{n}}{{\mathrm{\Delta }}{y}_{j}}+\displaystyle \frac{2{\xi }_{n}}{{\mathrm{\Delta }}{z}_{k}}+{{\rho }}_{e}\left(x,y,z\right){{\sigma }}_{C,\gamma }^{\mathrm{tot}}}.\end{eqnarray*}

**Figure 2.**
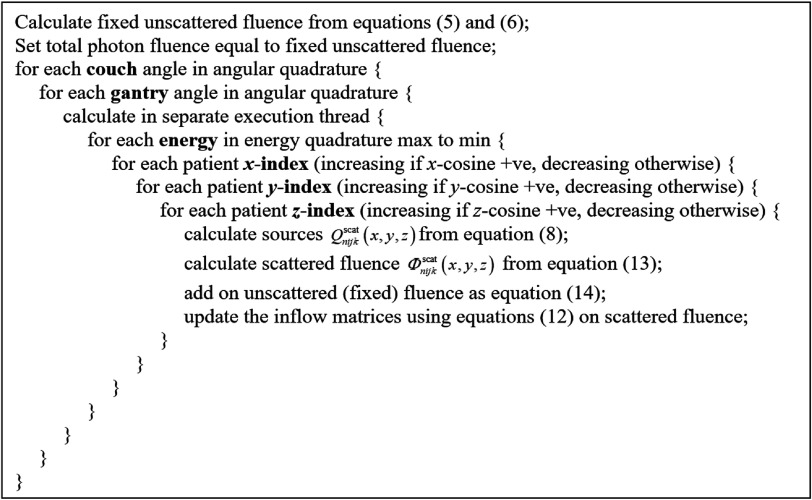
Transport sweep for photons.

As the unscattered fluence remained constant, regardless of the scattering events taking place, this fluence was added to the scattered fluence after each iteration of equation (13):

\begin{eqnarray*}{{\mathrm{\Phi }}}_{nijk}={{\mathrm{\Phi }}}_{nijk}^{\mathrm{scat}}+{{\mathrm{\Phi }}}_{nijk}^{\mathrm{unscat}},\end{eqnarray*}

where ${{\mathrm{\Phi }}}_{nijk}^{\mathrm{scat}}$ referred to the scattered fluence calculated from equation (13) and ${{\mathrm{\Phi }}}_{nijk}^{\mathrm{unscat}}$ referred to the unscattered fluence from equation (5) and (6). The overall procedure was as shown in the pseudocode of figure 2.

**Figure 3.**
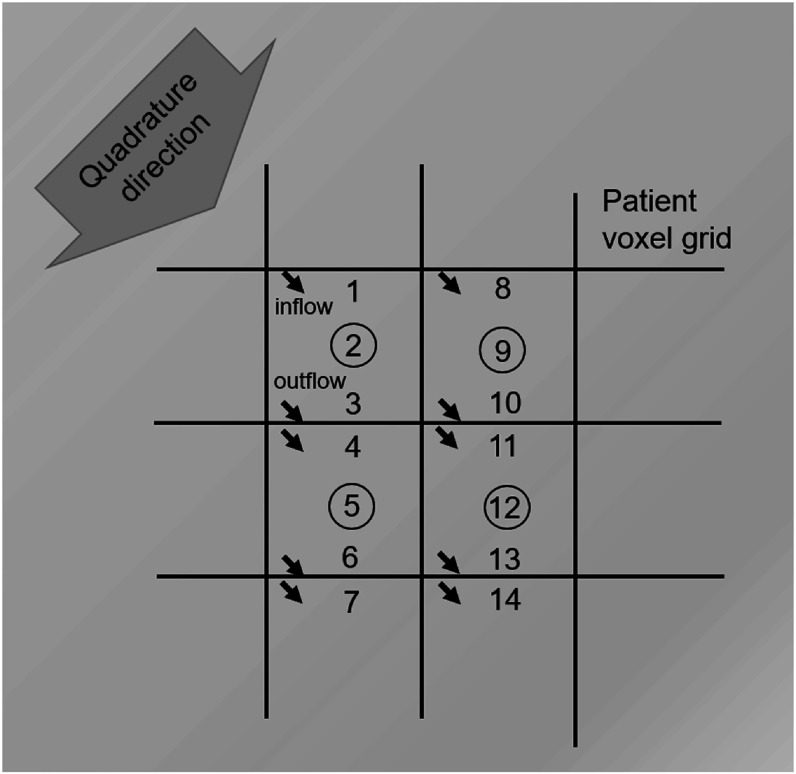
Schematic illustration of the transport sweep for one discrete direction and energy ordinate in relation to the patient voxel grid. Each directional sweep proceeded in the direction of decreasing particle energy, with the numerals indicating the order of calculation. For each voxel of the patient, the inflow was first calculated, then the fluence at the centre of the voxel (circled), which was the fluence actually used for the final dose calculation, then the outflow was calculated. The inflow for the next voxel was identical to the outflow for the current voxel.

Figure 3 also illustrates the process of performing the transport sweep. Multiple threads were used to carry out the sweeps for the different ordinates in parallel. Within each thread, the sweeps were ordered in descending order of photon energy and in the cardinal directions of the patient coordinate system, in the order ±*x*, ±*y*, ±*z*. Five iterations were found to be sufficient to give convergence of the photon equations, corresponding to five scattering events for each particle.

### 2.4. Electron transport

Once the photon distribution was defined, the Compton cross sections were used to calculate the fixed electron sources for electron transport. The electron fluence could then be calculated by solving the equations:

\begin{eqnarray*}\begin{array}{ccc}{{\boldsymbol{\Omega }}}_{e}\cdot {\mathrm{\nabla }}{{\mathrm{\Phi }}}_{e}\left({\bf{r}},{{\boldsymbol{\Omega }}}_{e},{E}_{e}\right) &amp; = &amp; {\rho }_{e}\left({\bf{r}}\right)\displaystyle {\int }_{0}^{\infty }\displaystyle {\int }_{4\pi }{\tilde{{\sigma }}}_{C,e}\left({E^{\prime} }_{\gamma },{E}_{e},{{\boldsymbol{\Omega }}{\boldsymbol{^{\prime} }}}_{\gamma }\cdot {{\boldsymbol{\Omega }}}_{e}\right){{\mathrm{\Phi }}}_{\gamma }\left({\bf{r}},{{\boldsymbol{\Omega }}{\boldsymbol{^{\prime} }}}_{\gamma },{E^{\prime} }_{\gamma }\right)d{{\boldsymbol{\Omega }}{\boldsymbol{^{\prime} }}}_{\gamma }d{E^{\prime} }_{\gamma }\\ &amp; &amp; +{\rho }_{e}\left({\bf{r}}\right)\displaystyle {\int }_{0}^{\infty }\displaystyle {\int }_{4\pi }{\tilde{\sigma }}_{M}\left({E^{\prime} }_{e},{E}_{e},{{\boldsymbol{\Omega }}{\boldsymbol{^{\prime} }}}_{e}\cdot {{\boldsymbol{\Omega }}}_{e}\right){{\mathrm{\Phi }}}_{e}\left({\bf{r}},{{\boldsymbol{\Omega }}{\boldsymbol{^{\prime} }}}_{e},{E^{\prime} }_{e}\right)d{{\boldsymbol{\Omega }}{\boldsymbol{^{\prime} }}}_{e}d{E^{\prime} }_{e}\\ &amp; &amp; +{\rho }_{c}\left({\bf{r}}\right)\displaystyle {\int }_{4\pi }{\sigma }_{\mathrm{Mott}}\left({\bf{r}},{E}_{e},{{\boldsymbol{\Omega }}{\boldsymbol{^{\prime} }}}_{e}\cdot {{\boldsymbol{\Omega }}}_{e}\right){{\mathrm{\Phi }}}_{e}\left({\bf{r}},{{\boldsymbol{\Omega }}{\boldsymbol{^{\prime} }}}_{e},{E}_{e}\right)d{{\boldsymbol{\Omega }}{\boldsymbol{^{\prime} }}}_{e}\\ &amp; &amp; -{\rho }_{e}\left({\bf{r}}\right){\sigma }_{M}^{\mathrm{tot}}\left({E}_{e}\right){{\mathrm{\Phi }}}_{e}\left({\bf{r}},{{\boldsymbol{\Omega }}}_{e},{E}_{e}\right)\\ &amp; &amp; -{\rho }_{c}\left({\bf{r}}\right){\sigma }_{\mathrm{Mott}}^{\mathrm{tot}}\left({\bf{r}},{E}_{e}\right){{\mathrm{\Phi }}}_{e}\left({\bf{r}},{{\boldsymbol{\Omega }}}_{e},{E}_{e}\right)\end{array}\end{eqnarray*}

where ${\rho }_{c}\left({\bf{r}}\right)$ was the density of atomic cores at position **r**, ${{\mathrm{\Phi }}}_{e}\left({\bf{r}},{{\boldsymbol{\Omega }}}_{e},{E}_{e}\right)$ was the electron fluence at position **r**, with direction ${{\boldsymbol{\Omega }}}_{e}$ and energy ${E}_{e},$ ${\tilde{{\sigma }}}_{C,e}\left({E^{\prime} }_{\gamma },{E}_{e},{{\boldsymbol{\Omega }}{\boldsymbol{^{\prime} }}}_{\gamma }\cdot {{\boldsymbol{\Omega }}}_{e}\right)$ was the differential Compton scattering cross section of a photon travelling initially with energy ${E^{\prime} }_{\gamma }$ in direction ${{\boldsymbol{\Omega }}{\boldsymbol{^{\prime} }}}_{\gamma }$ and giving rise to an electron travelling with energy ${E}_{e}$ and direction ${{\boldsymbol{\Omega }}}_{e},$ ${\tilde{{\sigma }}}_{M}\left({E^{\prime} }_{e},{E}_{e},{{\boldsymbol{\Omega }}{\boldsymbol{^{\prime} }}}_{e}\cdot {{\boldsymbol{\Omega }}}_{e}\right)$ was the differential Møller scattering cross section of an electron travelling initially with energy ${E^{\prime} }_{e}$ in direction ${{\boldsymbol{\Omega }}{\boldsymbol{^{\prime} }}}_{e}$ and finally with energy ${E}_{e}$ and direction ${{\boldsymbol{\Omega }}}_{e},$ ${\sigma }_{\mathrm{Mott}}\left({E}_{e},{{\boldsymbol{\Omega }}{\boldsymbol{^{\prime} }}}_{e}\cdot {{\boldsymbol{\Omega }}}_{e}\right)$ was the differential Mott scattering cross section of an electron travelling with energy ${E}_{e},$ initially in direction ${{\boldsymbol{\Omega }}{\boldsymbol{^{\prime} }}}_{e}$ and finally in direction ${{\boldsymbol{\Omega }}}_{e},$ ${\sigma }_{M}^{\mathrm{tot}}\left({E}_{e}\right)$ was the total Møller scattering cross section for an electron travelling initially with energy ${E}_{e},$ and ${\sigma }_{M\mathrm{ott}}^{\mathrm{tot}}\left({E}_{e}\right)$ was the total Mott scattering cross section for an electron travelling initially with energy ${E}_{e}$ (Hensel *et al* 2006).

The first integral of equation (15) was denoted by ${Q}_{nijk}^{\mathrm{fix}}$ and represented the fixed sources resulting from Compton interactions. The sum of three integrals was denoted by ${Q}_{nijk}.$ The differential scattering cross sections for electron scattering were singular at the angle of forward scattering, which was found to be problematic. Forward scattering was therefore excluded from equation (15) on the basis that a forward scattering event could be considered as a continuation of the particle fluence, so-called streaming, thereby not contributing to scatter. This was accomplished in practice in a similar manner to photons by zeroing the differential cross sections for $\theta \geqslant {\theta }_{0},$ where the value of *θ*_0_ was 1.0. The total scattering cross sections (which were almost completely dominated by the forward scattering direction) were adjusted accordingly, as follows:

\begin{eqnarray*}{{\sigma }}_{M}^{\mathrm{tot}}={{f}}_{M}{{\sigma }}_{M}^{0},\end{eqnarray*}

where ${{\sigma }}_{M}^{0}$ was the calculated theoretical total cross section for Møller scattering and *f*_M_ = 8.0 × 10^−5^ was the correction factor. Likewise, for Mott scattering:

\begin{eqnarray*}{{\sigma }}_{\mathrm{Mott}}^{\mathrm{tot}}\left(x,y,z\right)=\left({{f}}_{\mathrm{Mott},w}{{\sigma }}_{\mathrm{Mott}}^{0}\left(x,y,z\right)+{f}_{\mathrm{Mott},m}\displaystyle \frac{{A}_{w}-{A}_{m}\left(x,y,z\right)}{{N}_{A}}\right),\end{eqnarray*}

where ${{\sigma }}_{\mathrm{Mott}}^{0}\left(x,y,z\right)$ was the calculated theoretical total cross section for Mott scattering, *f*_Mott*,w*_ = 8.0 × 10^−3^ was a correction factor in water-equivalent material and *f*_Mott,*m*_ = 10.0 was a material correction factor, effective for materials of higher or lower density than water. This factor therefore varied with material type. The constant *A*_*w*_ was the atomic mass of water, *A*_*m*_ was the atomic mass of the material at location (*x*, *y*, *z*) and *N*_*A*_ was Avogadro’s number. This yielded a corrected total scatter cross section ${{\sigma }}_{\mathrm{Mott}}^{\mathrm{tot}}\left(x,y,z\right).$

The differential cross sections for Møller scattering were taken from equation 2.15 of ICRU37 (International Commission on Radiation Units and Measurements 1984), which was differential in energy, and then converted to differential in solid angle using the chain rule. The total Møller scattering cross section was calculated by integrating the differential cross section with respect to energy. The differential and total Mott scattering cross sections were taken from Hensel *et al* (2006), cross checked against ICRU28 (International Commission on Radiation Units and Measurements 1978) and also against the cross section differential in scattering angle cosine in the EGSnrc manual (Kawrakow *et al* 2019) with a change of variable to solid angle.

Unlike the photon equations, where the distribution of unscattered fluence was known beforehand and therefore excluded from the solution of equation (13), in the solution of the electron equations, the fixed sources were added to the other scatter sources in the right hand side of equation (15). Equation (15) was thus solved for the total electron fluence ${{\mathrm{\Phi }}}_{nijk}\left(x,y,z\right)$ by means of an analogous equation to equation (13):

\begin{eqnarray*}{{\mathrm{\Phi }}}_{nijk}=\displaystyle \frac{\displaystyle \frac{2{\mu }_{n}}{{\mathrm{\Delta }}{x}_{i}}{{\mathrm{\Phi }}}_{n,i-1/2,jk}+\displaystyle \frac{2{\eta }_{n}}{{\mathrm{\Delta }}{y}_{j}}{{\mathrm{\Phi }}}_{ni,j-1/2,k}+\displaystyle \frac{2{\xi }_{n}}{{\mathrm{\Delta }}{z}_{k}}{{\mathrm{\Phi }}}_{nij,k-1/2}+{Q}_{nijk}}{\displaystyle \frac{2{\mu }_{n}}{{\mathrm{\Delta }}{x}_{i}}+\displaystyle \frac{2{\eta }_{n}}{{\mathrm{\Delta }}{y}_{j}}+\displaystyle \frac{2{\xi }_{n}}{{\mathrm{\Delta }}{z}_{k}}+{{\rho }}_{e}\left(x,y,z\right){{\sigma }}_{M}^{\mathrm{tot}}+{{\rho }}_{c}\left(x,y,z\right){{\sigma }}_{\mathrm{Mott}}^{\mathrm{tot}}},\end{eqnarray*}

**Figure 4.**
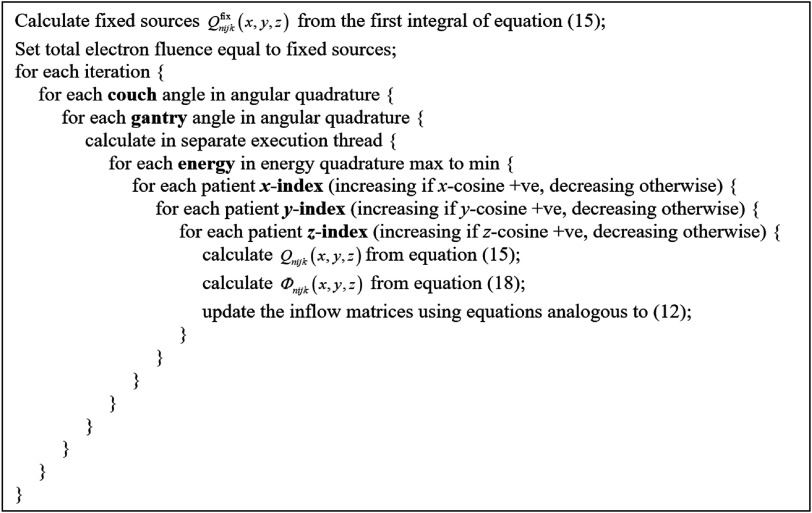
Transport sweep for electrons.

As with photons, the integrals of equation (15) were handled as discrete sums over the discrete ordinates, analogous to equation (9). Again, five transport sweeps were found to be sufficient for convergence. Figure 4 summarises the process.

### 2.5. Proton transport

For passive scattering, each beam was implicitly divided into 100 energy layers. The fluence weights, ${\eta }\left({E}_{p}\right),$ were determined using the formula of Bortfeld and Schlegel (1996) for discrete energy layers, subsequently corrected by a factor ${\left(d/R\right)}^{0.7},$ where *d* was the depth and *R* was the nominal range of the energy in question. This corrected formula was found to be more accurate than the original formula when using real depth-dose curves rather than the simple analytical curves described by Bortfeld (1997). These weights were then converted to monitor units by allowing for the in-air stopping power of the protons in the monitor chamber (Sahoo *et al* 2008, Zhu *et al* 2010, Clasie *et al* 2012). With proton arcs, the arc segments were collected into groups, each group representing 20° of gantry arc, and the individual segments within each group were assigned specific energies, thereby forming energy layers.

The three types of interactions of protons with matter (Newhauser and Zhang 2015) were dealt with as follows. Due to the high mass of protons in comparison with electrons (1832 times the mass), the protons were considered to continue in approximately the same direction during inelastic Coulomb interactions, so that the process could be represented by a continuous slowing down approximation (CSDA). Inelastic nuclear interactions were represented as a linear loss of proton fluence in the fixed sources (Bortfeld 1997). Consequently the proton fluence in the presence of these two types of interaction could be handled as a fixed source for purposes of solving the LBTEs. The LBTE were then used to solve for elastic Coulomb interactions with the atomic nucleus, Mott scattering. Note that range straggling was considered in this approach to be a multiple-scattering effect, so was not included in the fixed CSDA sources, but resulted naturally from the LBTE solution.

For each of the 100 initial energies contributing to the spread-out Bragg peak, the energy with depth was calculated recursively as:

\begin{eqnarray*}e\left({{\bf{r}}}_{n+1}\right)=e\left({{\bf{r}}}_{n}\right)-\left|{{\bf{r}}}_{n+1}-{{\bf{r}}}_{n}\right|\rho \left({{\bf{r}}}_{n}\right)S\left(e\left({{\bf{r}}}_{n}\right),{\rho }_{e}\left({{\bf{r}}}_{n}\right),\rho \left({{\bf{r}}}_{n}\right)\right),\end{eqnarray*}

where *n* indexed the voxels traced by the protons, ${{\bf{r}}}_{n}$ represented the position vector of voxel *n*, $e\left({{\bf{r}}}_{n}\right)$ was the energy of the protons at that location, $\left|{{\bf{r}}}_{n+1}-{{\bf{r}}}_{n}\right|$ was the equivalent path length of the protons between voxels indexed *n* and *n*+1. $\rho \left({{\bf{r}}}_{n}\right)$ was the mass density at voxel *n* and ${\rho }_{e}\left({{\bf{r}}}_{n}\right)$ was the electron density. The corresponding fluence was calculated as:

\begin{eqnarray*}{{\mathrm{\Phi }}}_{p}^{\mathrm{unscat}}\left({\bf{r}},{E}_{p}\right)=\displaystyle \frac{1}{{z}^{2}}\left({\psi }\left(x,y\right)\otimes s\left(x,y\right)\right){\eta }\left({E}_{p}\right)\left(1-{\lambda }z-\tau {\sigma }_{\mathrm{Mott}}^{\mathrm{tot}}\left({\bf{r}},{E}_{p}\right)\right),\end{eqnarray*}

where the notation was the same as in equation (5). The constant *λ* = 5 × 10^−4^ represented the loss of proton fluence per unit radiological path length due to nuclear interactions and the constant *τ* = 1.0 × 10^−3^ represented the loss of fluence due to elastic scattering. As with electron transport, singularities in the Mott cross sections were handled by zeroing the cross section for scattering angles with cosine greater than or equal to a threshold, *θ*_0_ = 1.0, and then reducing the total cross section accordingly. This latter adjustment was carried out in an energy-dependent manner:

\begin{eqnarray*}{\sigma }_{\mathrm{Mott}}^{\mathrm{tot}}\left({\bf{r}},{E}_{p}\right)={\sigma }_{\mathrm{Mott}}^{0}\left({\bf{r}},{E}_{p}\right)\left[\left(\frac{E-e}{E}\right){f}_{\mathrm{low}}+\frac{e}{E}{f}_{\mathrm{high}}\right],\end{eqnarray*}

where ${\sigma }_{\mathrm{Mott}}^{0}\left({\bf{r}},{E}_{p}\right)$ was the calculated theoretical total cross section, *e* = 0…*E*-1 was the index of the energy quadrature, *f*_low_ = 0.8 was the low-energy reduction factor and *f*_high_ = 0.05 was the high-energy reduction factor.

The reduction in fluence represented by the final term in equation (20) then formed scattered fluence, for which the LBTE were as follows:

\begin{eqnarray*}\begin{array}{c}{{\boldsymbol{\Omega }}}_{p}\cdot {\mathrm{\nabla }}{{\mathrm{\Phi }}}_{p}^{\mathrm{scat}}\left({\bf{r}},{{\boldsymbol{\Omega }}}_{p},{E}_{p}\right)={\rho }_{c}\left({\bf{r}}\right)\displaystyle {\int }_{4\pi }{\sigma }_{\mathrm{Mott}}\left({\bf{r}},{E}_{p},{{\boldsymbol{\Omega }}{\boldsymbol{^{\prime} }}}_{p}\cdot {{\boldsymbol{\Omega }}}_{p}\right){{\mathrm{\Phi }}}_{p}^{\mathrm{scat}}\left({\bf{r}},{{\boldsymbol{\Omega }}{\boldsymbol{^{\prime} }}}_{p},{E}_{p}\right)d{{\boldsymbol{\Omega }}{\boldsymbol{^{\prime} }}}_{p}\\ -{\rho }_{c}\left({\bf{r}}\right){\sigma }_{\mathrm{Mott}}^{\mathrm{tot}}\left({\bf{r}},{E}_{p}\right){{\mathrm{\Phi }}}_{p}^{\mathrm{scat}}\left({\bf{r}},{{\boldsymbol{\Omega }}}_{p},{E}_{p}\right),\end{array}\end{eqnarray*}

and the integral term was denoted as ${Q}_{nijk}^{\mathrm{scat}}.$ The LBTE were then solved iteratively in the same manner as for photons in equations (13) and (14):

\begin{eqnarray*}{{\mathrm{\Phi }}}_{nijk}=\displaystyle \frac{\displaystyle \frac{2{\mu }_{n}}{{\mathrm{\Delta }}{x}_{i}}{{\mathrm{\Phi }}}_{n,i-1/2,jk}^{\mathrm{scat}}+\displaystyle \frac{2{\eta }_{n}}{{\mathrm{\Delta }}{y}_{j}}{{\mathrm{\Phi }}}_{ni,j-1/2,k}^{\mathrm{scat}}+\displaystyle \frac{2{\xi }_{n}}{{\mathrm{\Delta }}{z}_{k}}{{\mathrm{\Phi }}}_{nij,k-1/2}^{\mathrm{scat}}+{Q}_{nijk}^{\mathrm{scat}}}{\displaystyle \frac{2{\mu }_{n}}{{\mathrm{\Delta }}{x}_{i}}+\displaystyle \frac{2{\eta }_{n}}{{\mathrm{\Delta }}{y}_{j}}+\displaystyle \frac{2{\xi }_{n}}{{\mathrm{\Delta }}{z}_{k}}+{{\rho }}_{e}\left(x,y,z\right){{\sigma }}_{C,\gamma }^{\mathrm{tot}}}+{{\mathrm{\Phi }}}_{nijk}^{\mathrm{unscat}}.\end{eqnarray*}

### 2.6. Absorbed dose

Dose was calculated by summing the electron fluence (in the case of photon beams) or proton fluence (in the case of proton beams) from all discrete ordinates at a given energy, and then multiplying by the mass collision stopping power for that energy (Larsen *et al* 1997, Ma and Li 2011). For photon beams:

\begin{eqnarray*}D\left({\bf{r}}\right)=\displaystyle \frac{1}{\rho \left({\bf{r}}\right)}\displaystyle {\int }_{0}^{\infty }\displaystyle {\int }_{4\pi }{S}_{e}\left({\bf{r}},{E^{\prime} }_{e}\right){{\mathrm{\Phi }}}_{e}\left({\bf{r}},{{\boldsymbol{\Omega }}{\boldsymbol{^{\prime} }}}_{e},{E^{\prime} }_{e}\right)d{\boldsymbol{\Omega }}{\boldsymbol{^{\prime} }}d{E^{\prime} }_{e},\end{eqnarray*}

which, in the discrete context of the calculations, was approximated as:

\begin{eqnarray*}D\left({\bf{r}}\right)=\displaystyle \frac{1}{\rho \left({\bf{r}}\right)}\displaystyle \sum _{e=1}^{{N}_{e}}{S}_{e}\left({\bf{r}},{E}_{e}\right)\displaystyle \sum _{n=1}^{{N}_{a}}{{\mathrm{\Phi }}}_{e}\left({\bf{r}},{{\boldsymbol{\Omega }}}_{e},{E}_{e}\right).\end{eqnarray*}

Similarly, for protons:

\begin{eqnarray*}D\left({\bf{r}}\right)=\displaystyle \frac{1}{\rho \left({\bf{r}}\right)}\displaystyle \sum _{e=1}^{{N}_{e}}{S}_{p}\left({\bf{r}},{E}_{e}\right)\displaystyle \sum _{n=1}^{{N}_{a}}{{\mathrm{\Phi }}}_{p}\left({\bf{r}},{{\boldsymbol{\Omega }}}_{p},{E}_{p}\right).\end{eqnarray*}

The mass collision stopping power, *S*_*e*_, was obtained from the formula given in ICRU37 (equations 2.16 and 2.17) (International Commission on Radiation Units and Measurements 1984) with a correction for density effect according to Sternheimer *et al* (1983) For simplicity, the material for the Sternheimer density effect was taken to be water throughout.

For all of the dose calculation, the stoichiometric tissue conversion (Schneider *et al* 1996, 2000) of Vanderstraeten *et al* (2007) was used. Mass density was calculated using a lookup table for the CT scanner in use. The Hounsfield numbers were used to categorise the tissue into one of 14 ranges, each range providing the relative proportion by mass of 12 elements. The atomic numbers and weights of these elements and their relative proportions were then used to calculate the electron and nuclear densities at each voxel and the scattering cross sections.

### 2.7. Inclusion of spatial uncertainty

A simple means of robust treatment planning (Unkelbach *et al* 2018) was incorporated by considering the clinical target volume (CTV) to have a spatial probability distribution (Bedford *et al* 2019). In practice, this was carried out by shifting the unscattered fluence in the opposite direction to the direction of target volume motion. In each of the three orthogonal Cartesian directions in the patient coordinate system, a set of positions was specified, ranging from −16 to +16 mm in 2 mm steps, relative to the planned CTV position. The operator then specified the probability of finding the CTV at each of these coordinates. The probability of finding the CTV at coordinate (*x*_*i*_, *y*_*j*_, *z*_*k*_) was therefore given by:

\begin{eqnarray*}p\left({x}_{i},{y}_{j},{z}_{k}\right)=p\left({x}_{i}\right)p\left({y}_{j}\right)p\left({z}_{k}\right),\end{eqnarray*}

and a discrete uncertainty kernel was therefore constructed as a series of coordinates (*x*_*i*_, *y*_*j*_, *z*_*k*_) with corresponding probabilities $p\left({x}_{i},{y}_{j},{z}_{k}\right).$ As this led to a combination of 4913 points, which was computationally demanding, the kernel was resampled by casting the points onto a regular grid of resolution 2 mm and then selecting the voxels with the highest 64 intensities, with rescaling to ensure conservation of energy (Bedford *et al* 2019). The result was a kernel consisting of 64 coordinates and probabilities. Unscattered fluence was then distributed according to the relation:

\begin{eqnarray*}{{\mathrm{\Phi }}}_{ijk}^{\mathrm{unscat}}\left(x+{x}_{i},y+{y}_{j},z+{z}_{k}\right)=p\left({x}_{i},{y}_{j},{z}_{k}\right){{\mathrm{\Phi }}}^{\mathrm{unscat}}\left(x,y,z\right),\end{eqnarray*}

where ${{\mathrm{\Phi }}}^{\mathrm{unscat}}$ referred to each of the four fluences in equation (6) in turn. Thus, the calculated fluence of equation (5) (for photon beams) or (20) (for proton beams) was distributed over the appropriate angular ordinates of equation (6) and voxels of equation (28). Note that this approach produced a single expectation value for the dose distribution, as opposed to a range of discrete scenarios, which would take too long to calculate in the present context.

### 2.8. Adaptive patient model

The LBTE were solved on a regular Cartesian grid of resolution 2.5 mm × 2.5 mm × 2.5 mm. The dose calculation grid itself was adaptive. The unscattered fluence within each cluster of 4 × 4 × 4 voxels was evaluated and if less than a given percentage of the maximum unscattered fluence, that cluster of voxels was combined into a single voxel of dimensions 10 mm × 10 mm × 10 mm. For photons, the threshold was 20% and for protons, the threshold was 5%. The accuracy of calculation was not very sensitive to the choice of threshold, but selecting a threshold too low caused high dose resolution to be used throughout the patient, thereby taking additional calculation time, and a value too high caused that the low resolution was used too close to the high-dose region, thereby affecting the visualisation of dose fall-off. This latter situation was prone to occur with protons at the distal edge of the spread-out Bragg peak, so the lower threshold of 5% was used with protons. The inflowing fluences to such an adaptive voxel, i.e. ${{\mathrm{\Phi }}}_{n,i-1/2,jk},$ ${{\mathrm{\Phi }}}_{ni,j-1/2,k}$ and ${{\mathrm{\Phi }}}_{nij,k-1/2}$ were calculated by taking the mean of the outgoing fluences from the previous 16 separate voxels abutting this voxel. The outgoing fluences from the adaptive voxel, i.e. ${{\mathrm{\Phi }}}_{n,i+1/2,jk}$, ${{\mathrm{\Phi }}}_{ni,j+1/2,k}$ and ${{\mathrm{\Phi }}}_{nij,k+1/2}$ in equation (12), to the 16 following voxels were all replicated from the single value calculated for the large adaptive voxel. In the event that multiple adaptive voxels were adjacent, which was commonly the case, the transport proceeded normally between them as in the normal voxels.

### 2.9. Implementation

The above scheme was incorporated into a simple evaluation environment enabling the visualisation of the particle fluences and the resulting absorbed dose. It was also operated as a dose calculation in the AutoBeam (v6.0) in-house treatment planning system (Bedford 2009, 2013). The software was written in multithreaded Java for speed and cross-platform portability, and was operated on a SPARC T4 server (Sun Microsystems, Oracle Corporation, Reading, UK) with 128 hyperthreads and 128 GB memory.

The eventual goal of this work was to use the LBTE solver in the context of the inverse planning optimiser. For photons, the dose distributions were therefore compared with those produced by the fast convolution algorithm already implemented in the AutoBeam environment (Bedford 2002). The convolution algorithm had previously been validated and used routinely for production of clinical VMAT plans (Bedford *et al* 2008). For protons, the comparison algorithm was a modified version of the analytical algorithm by Bortfeld (Bortfeld and Schlegel 1996, Bortfeld 1997). In particular, range straggling was included at radiological depth *r* by averaging three dose values:

\begin{eqnarray*}D\left(r\right)=\left[d\left(r-{\mathrm{\Delta }}\right)+d\left(r\right)+d\left(r+{\mathrm{\Delta }}\right)\right]/3,\end{eqnarray*}

where $D\left(r\right)$ was the total dose and $d\left(r\right)$ was the value using the method described by Bortfeld (Bortfeld and Schlegel 1996, Bortfeld 1997). The value of Δ was chosen empirically as 1.0 mm.

### 2.10. Validation

Basic validation was performed by comparing depth doses and profiles between the LBTE solver and convolution in the case of photon beams and between the LBTE solver and the analytical algorithm in the case of proton beams. For photons, this was performed for a 100 mm × 100 mm photon beam with 1000 mm source-to-axis distance and 850 mm source-to-surface distance. For protons, a 100 mm × 100 mm passively scattered beam was used with 2300 mm source-to-axis distance and 2150 mm source-to-surface distance. The proton beam energy ranged from 115 to 174 MeV so as to provide a spread-out Bragg peak from 100 to 200 mm depth. Field size tests using fields from 20 mm × 20 mm to 200 mm × 200 mm were also performed but are not described here in the interests of brevity. Likewise, a 100 mm × 100 mm × 300 mm inhomogeneity was introduced into a 300 mm × 300 mm × 300 mm phantom and assigned densities from 0 g cm^−3^ (vacuum) through 0.23 g cm^−3^ (lung), 1.51 g cm^−3^ (dense bone) to 4.50 g cm^−3^ (titanium) to assess the dose calculation in heterogeneous media, but these tests are not discussed further. In the presentation of results, absorbed dose was normalised such that the overall maximum dose in the complete three-dimensional LBTE solution was designated 100% and the convolution or analytical solution was normalised by the same factor to ensure preservation of the relative magnitude of the two methods.

In order to ensure that the scheme was converging completely, the evolution of particle fluence was observed for each type of particle: photons, electrons and protons. The fluence directions at 0° and 90° to the beam axis of a single 100 mm × 100 mm beam were chosen for this purpose. The beam was directed to a water-equivalent phantom of dimensions 300 mm × 300 mm × 300 mm, and also to a lung-equivalent phantom (mass density 0.25 g cm^−3^) and a bone-equivalent phantom (mass density 1.3 g cm^−3^), so as to consider the main types of tissue likely to be encountered practically. The dose calculation was run for 10 iterations in each case, and the fluence relative to that of the final iteration was examined at the centre of the beam and phantom, 150 mm deep. In the case of the proton beam, the beam energy spectrum was varied according to the material density so as to maintain a spread-out Bragg peak from 100 to 200 mm depth.

To assess the adequacy of the relatively simple angular quadrature used in this work, the dose was calculated using the quadrature described above (section 2.2) and then compared with that calculated using a more comprehensive quadrature. This consisted of the same coordinate system as in section 2.2 but used ordinates in both couch and gantry angle at 10° separation. The ordinates were weighted according to the solid angle that they occupied in angular space (see figure 1). Thus, they were weighted equally in couch angle but weights in gantry angle were set according to:

\begin{eqnarray*}w\left(c,g\right)={w}_{0}\,\sin \left(g\right),\end{eqnarray*}

where *c* and *g* were the couch and gantry angles, in radians, of the ordinates, respectively, and *w*_0_ was the solid angle subtended by the ordinates with gantry angle $\pi /2:$

\begin{eqnarray*}{w}_{0}=\displaystyle \frac{2{\pi }^{2}}{CG},\end{eqnarray*}

where *C* (=18) was the number of couch ordinates and *G* (=36) was the number of gantry ordinates. With the more comprehensive quadrature, the remaining details were identical to those given above, except for using *θ*_0_ = 0.97, ${f}_{C,\gamma }$= 7.5 and ${f}_{C,\gamma }^{\mathrm{tot}}$ = 3.5 for photons, *θ*_0_ = 0.97, *f*_M_ = 4.0 × 10^−4^ and *f*_Mott*,w*_ = 4.0 × 10^−3^ for electrons and *θ*_0_ = 0.93, *λ* = 2.0 × 10^−4^ and *τ* = 1.5 × 10^−3^ for protons.

### 2.11. Treatment plans

Finally, the performance of the LBTE solver was evaluated by producing treatment plans for a demanding tumour environment. This typically occurs in the treatment planning of lung patients, so the CT scan of a lung patient from a clinical trial of SPECT lung perfusion in radiotherapy (Weller *et al* 2019) was retrospectively used for this study. The margin from the gross tumour volume (GTV) to the CTV was 5 mm. A further margin of 5 mm from the CTV to the planning target volume (PTV) was used. The PTV had a volume of 95.7 cm^3^.

Three types of treatment plan were produced: photon VMAT, proton passive scattering and proton arcs. The dose prescription was a median of 64 Gy in 32 fractions to the PTV. The isocentre was positioned at the centre of the PTV and a single coplanar counterclockwise arc was used to create the photon treatment plan. The gantry angle ranged from 179° to 339° i.e. 200° of arc with 4° control point spacing, giving 51 control points. Collimator angle was fixed at 2°. For the passive scattering proton treatment plans, four fixed beams were used, for delivery with passive scattering. Gantry angles were 135°, 90°, 45° and 0°. In this case, the range of energies used for each beam was calculated according to the minimum and maximum equivalent path length encountered across the PTV, and a range shifter was used to adapt the isodoses to the distal contour of the PTV. For proton arc planning, the same gantry angles were used as for photons. The plans were created using the standard algorithms available within AutoBeam (see section 2.9) and recalculated using LBTE for comparison purposes. The plans were evaluated using dose–volume histograms.

The general performance of the LBTE algorithm was also assessed by comparing LBTE with Monte Carlo simulation for a prostate plan and a lung plan, both plans using photon IMRT. These plans were previously used for a comparison of tumour tracking technologies and the Monte Carlo dose distributions were also previously reported (Bedford *et al* 2022). Further details of the plans and the phase space model are given in that publication.

## 3. Results

### 3.1. Basic validation

**Figure 5.**
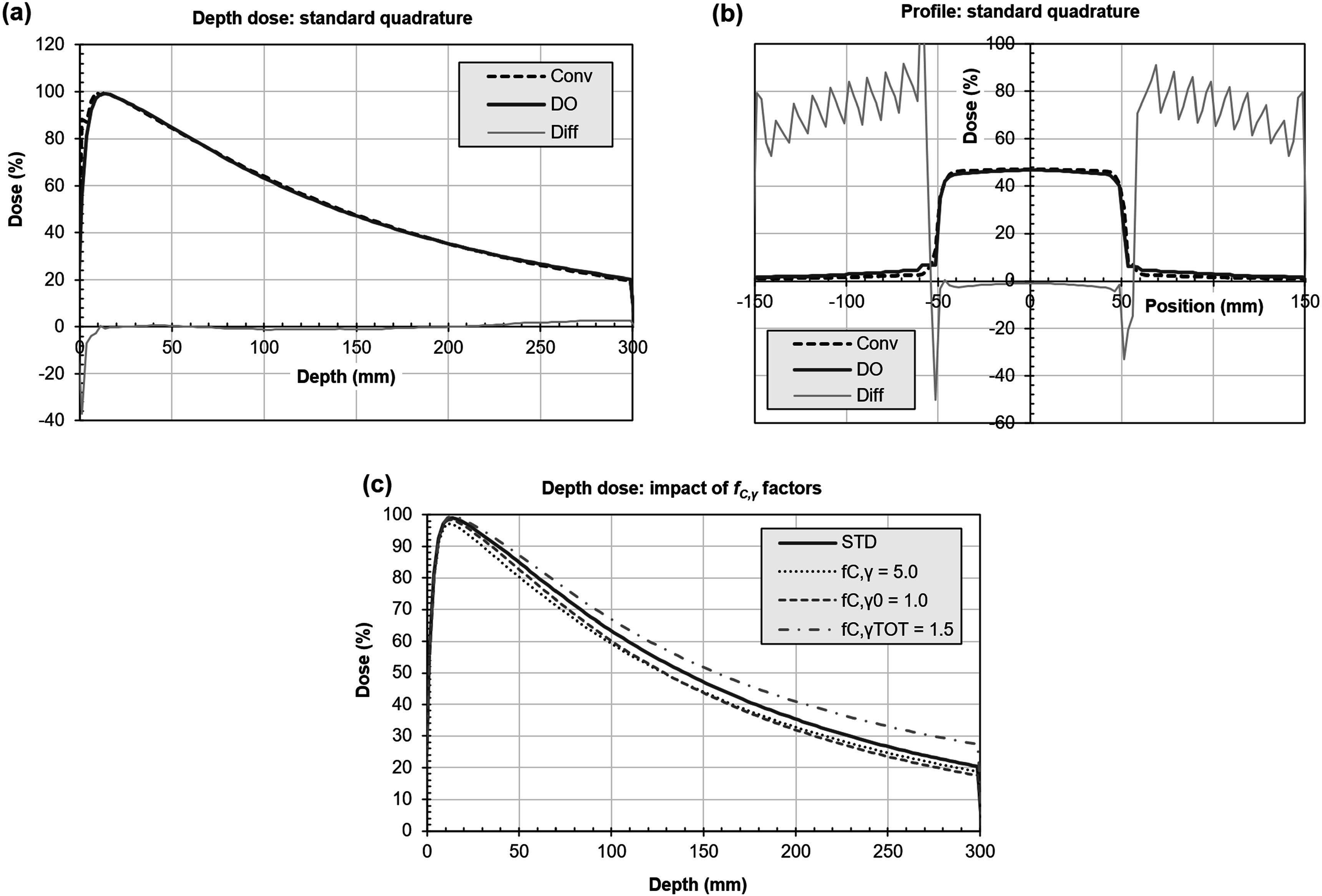
Photon validation. (a) Depth dose and (b) profile at isocentric depth 150 mm for discrete ordinates (DO) and convolution (Conv). The difference between the two curves as a percentage of local dose is also shown (Diff). (Some of the percentage differences at low dose are high so the scale is not extended to include them.) (c) Depth dose showing the impact of varying ${f}_{C,\gamma },$ ${f}_{C,\gamma }^{0}$ and ${f}_{C,\gamma }^{\mathrm{tot}}.$ Each factor is reduced by 50% in turn and compared against the standard set of factors ${f}_{C,\gamma }$= 10.0, ${f}_{C,\gamma }^{0}$ = 2.0, ${f}_{C,\gamma }^{\mathrm{tot}}$ = 3.0 (STD, same curve as DO in (a)).

Examples of the agreement between the dose distributions produced by the LBTE solver and convolution are shown for a photon beam in figure 5. The agreement between the two methods is generally within 2% except in the high-dose gradients and out-of-field region. The convolution approach has limited accuracy in the buildup region due to its discrete scatter kernel and in the out-of-field region due to its use of a uniform transmission value (Bedford 2002). Figure 5(c) also shows the effect of varying the parameters ${f}_{C,\gamma }^{0},$ ${f}_{C,\gamma }$ and ${f}_{C,\gamma }^{\mathrm{tot}}$ by a factor of two. These factors principally affect the gradient and curvature of the depth-dose curve. The dose distribution is not very sensitive to these parameters.

**Figure 6.**
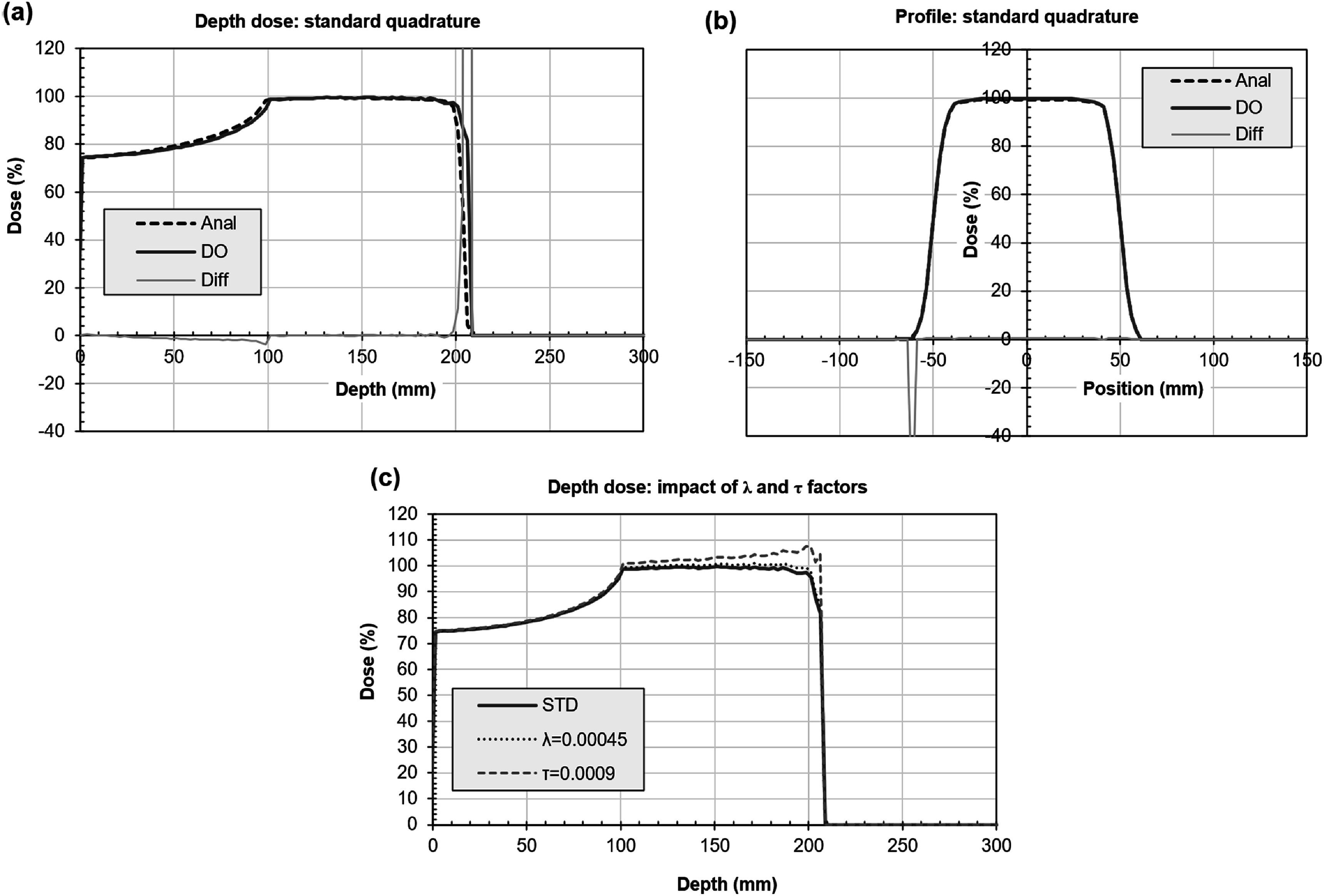
Proton validation. (a) Depth dose and (b) profile at isocentric depth 150 mm for discrete ordinates (DO) and an analytical method (Anal). The difference between the two curves as a percentage of local dose is also shown (Diff). (Some of the percentage differences at low dose are high so the scale is not extended to include them.) (c) Depth dose showing the impact of varying *λ* and *τ* of equation (20). Each factor is reduced by 10% in turn and compared against the standard set of factors *λ* = 0.0005 and *τ* = 0.001 (STD, same curve as DO in (a)).

The corresponding comparison for proton beams is shown in figure 6. In general, the agreement is within 2% with the exception of the distal edge of the spread-out Bragg peak. Here the range of the beam varies by approximately 3 mm between the two calculations, which could be clinically significant, although both methods attain zero at the same depth. The difference in range is therefore due to a difference in steepness of the distal edge, the accuracy of which is limited by the 2.5 mm voxel size. The impact of varying factors *λ* and *τ* of equation (20) is also shown in figure 6(c). The factor *λ* has a gentle effect along the whole of the depth dose curve, where non-elastic scattering takes place, whereas *τ* has a preferential effect near to the distal edge of the depth dose, where elastic scattering is dominant. The dose distribution is comparatively sensitive to these factors, but they are robust. In other words, once chosen, they are suitable for use in a variety of beam sizes and materials.

Computation time is in the order of 2–5 min for calculation of a photon beam or proton beam, depending on the dose grid size and resolution. There is a slight dependency on the number of beams and segments in the treatment plan being calculated due to the time taken to calculate fixed sources, but the overall time is almost unaffected as the transport sweeps are similar, regardless of the initial unscattered fluence.

### 3.2. Convergence

**Figure 7.**
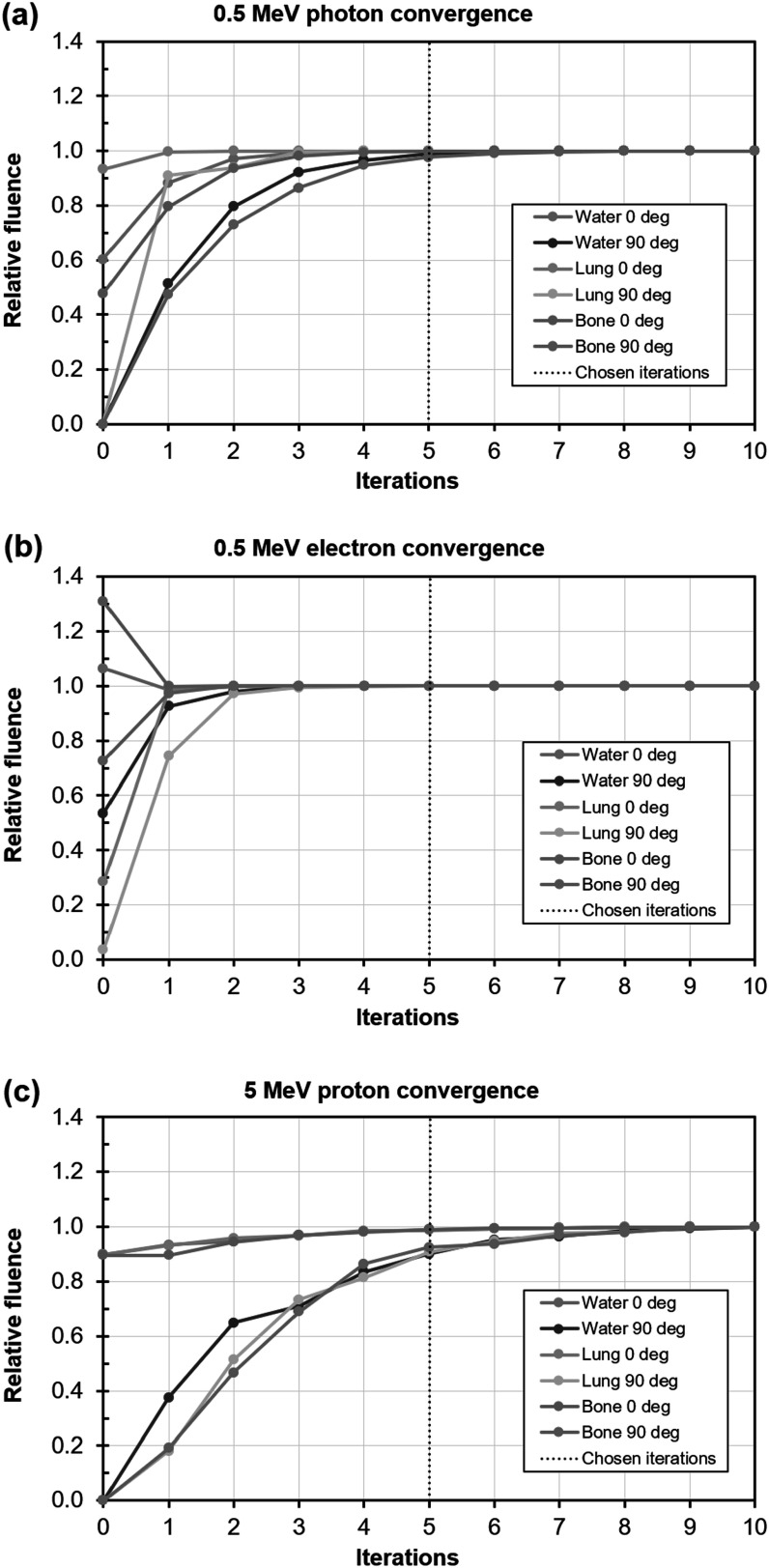
Examples of convergence for photons, electrons and protons. The chosen number of five iterations is shown in each case by the dotted line.

The convergence of the discrete ordinates scheme is shown in figure 7 for photons, electrons and protons in several materials. In general, the development of fluence at 90° to the beam requires more iterations than development of fluence at 0° to the beam due to the larger number of scattering events required for the larger angle. With photons, a higher-density material takes longer to converge, with electrons, a lower-density material takes longer to converge, and with protons, the density has little effect on convergence. Note that at gantry 90°, the fluence is approximately one order of magnitude lower than at 0° for all of the types of transport, thereby having a correspondingly small impact on final computed dose. Thus, in the case of protons, convergence of the 90° fluence to 90% of its final value is sufficient to provide absorbed dose within 1% of the converged value. Continuing to iterate for longer is undesirable in terms of computational time, particularly in the inverse planning context.

### 3.3. Angular quadrature

**Figure 8.**
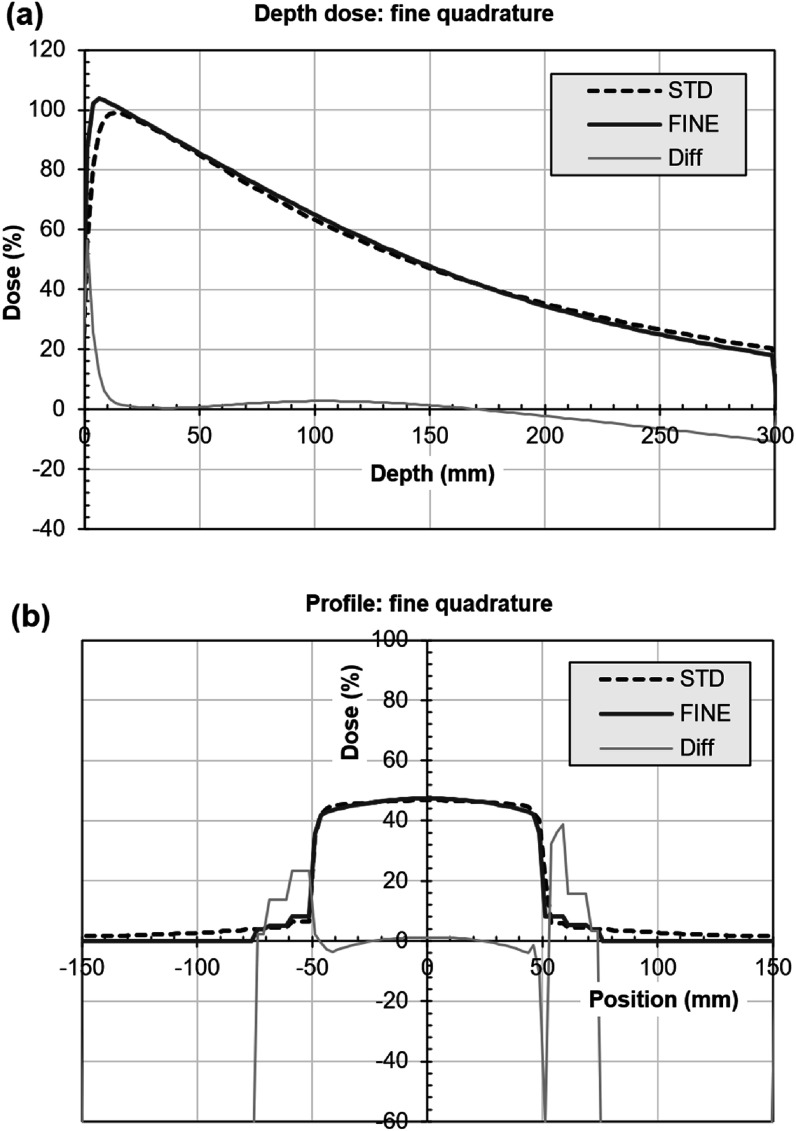
Increased number of angular ordinates in calculation of a photon beam. (a) Depth dose and (b) profile at isocentric depth 150 mm for discrete ordinates using fine (FINE) or standard (STD) quadrature. The difference between the two curves as a percentage of local dose is also shown (Diff). (Some of the percentage differences at low dose are high so the scale is not extended to include them.) The differences in the profile (b) beyond ±75 mm are due to the use of a narrower phantom for the fine quadrature.

Figure 8 shows the depth dose curve and profile when the number of angular ordinates is increased. Generally the result is very similar to when using the standard angular quadrature. Some differences are observed around the depth of maximum dose in the depth dose curve and in the in-field region of the profile. The out-of-field differences in the profile are considered to be primarily due to the limited accuracy of the convolution model. The calculation time in this case was around 18 h, the increased number of angular ordinates having a considerable impact on computational time and memory resources used.

**Figure 9.**
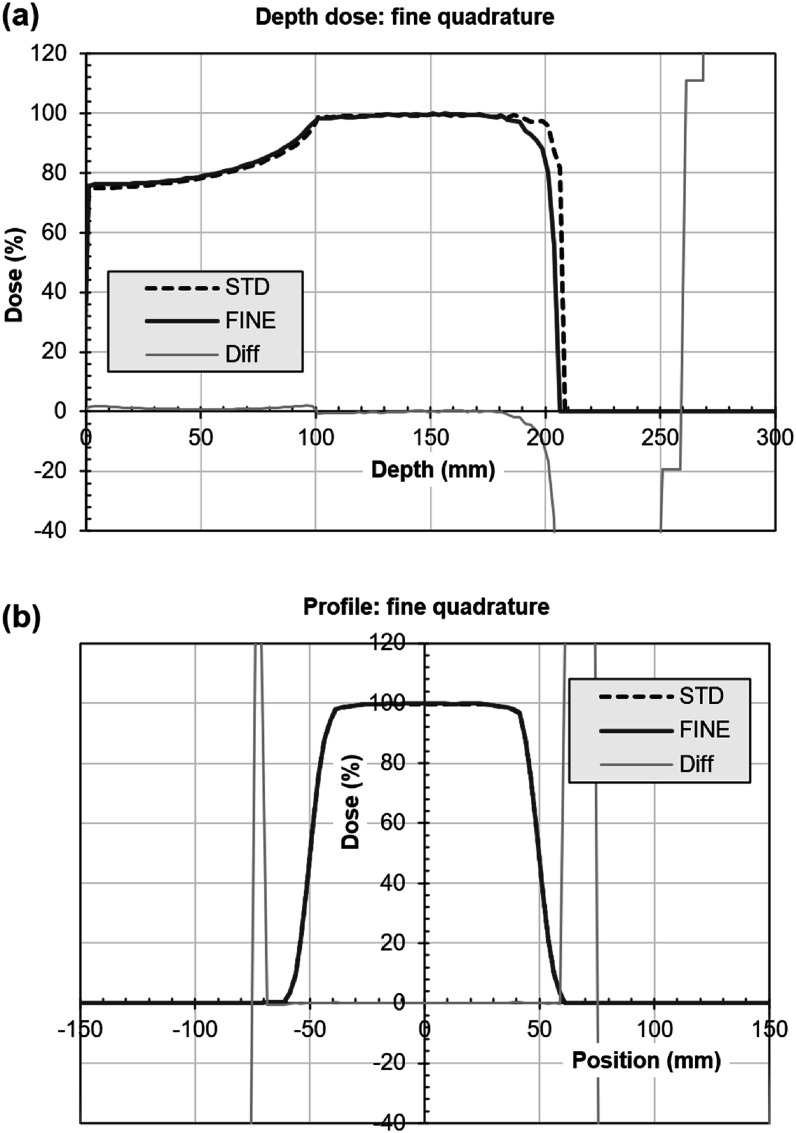
Increased number of angular ordinates in calculation of a proton beam. (a) Depth dose and (b) profile at isocentric depth 150 mm for discrete ordinates using fine (FINE) or standard (STD) quadrature. The difference between the two curves as a percentage of local dose is also shown (Diff) (some of the percentage differences at low dose are high so the scale is not extended to include them).

Figure 9 shows the corresponding comparison for a proton beam. The proton range with fine quadrature is slightly lower than with standard quadrature, so that the overestimation of range seen in figure 6 is corrected. The resulting distal edge of the spread-out Bragg peak for the LBTE solution is in good agreement with that of the analytical model, except for a difference of around 5% in the final 10 mm of the high-dose region. The profiles produced by the LBTE and analytical models are within 1%.

### 3.4. Lung treatment plans

**Figure 10.**
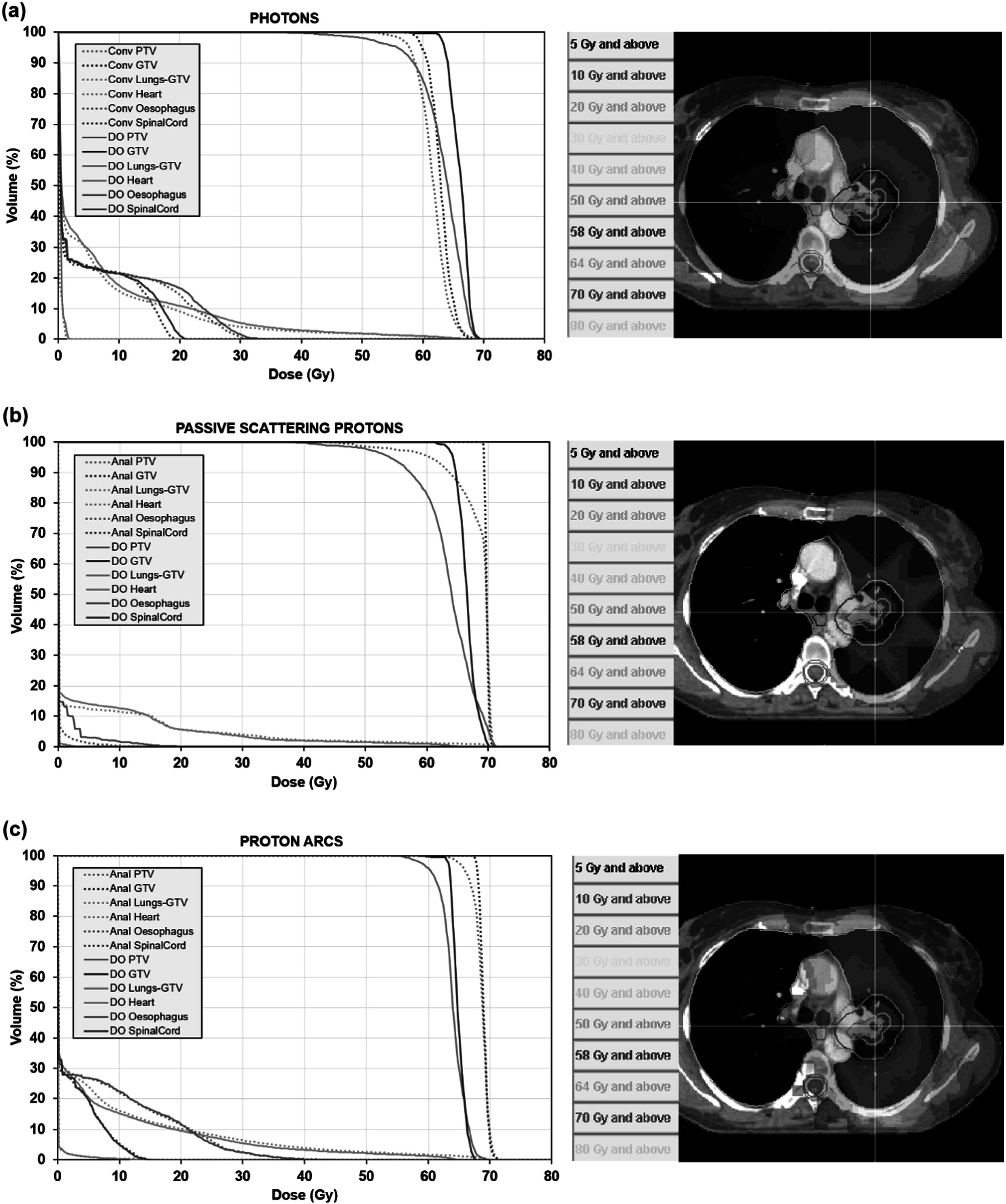
Cumulative dose–volume histograms for the lung treatment plans using (a) photon VMAT, (b) passive scattered protons, and (c) proton arcs, with discrete ordinates calculation (DO, solid lines) and convolution (Conv, dotted lines) or an analytical method (Anal, dotted lines). The discrete ordinates plans are prescribed such that the PTV median dose is 64 Gy in 32 fractions, and the convolution and analytical plans are calculated for the same monitor units as the discrete ordinates plans. The transaxial discrete ordinates dose distribution is shown on the right for each type of treatment plan.

Dose–volume histograms for the photon treatment plan, as calculated by convolution and discrete ordinates, are shown in figure 10(a). The inset to this figure shows the transaxial dose distribution calculated by discrete ordinates. The doses to the organs at risk are similar for the two calculation methods, but the dose to the GTV and PTV vary. The convolution method shows good homogeneity in target dose, but the coverage is found to be not so high when the final dose is recalculated with the Boltzmann solver. This is a result of the more comprehensive treatment of the inhomogeneities by the Boltzmann solver, in comparison to the use of spatially invariant scatter kernels in the convolution calculation.

Similar results are observed in the dose–volume histograms for passively scattered protons (figure 10(b)) as with photons. In this case, the comparison calculation is relatively simple, so the PTV dose appears very homogeneous centrally, with a loss of dose peripherally due to the motion modelling. When the final dose calculation is carried out with the Boltzmann solver, the PTV dose distribution is different, with much of the GTV and PTV receiving a lower dose.

With proton arcs (figure 10(c)), the effect is also seen. The simple algorithm produces a homogeneous dose distribution in the PTV, but the heterogeneity is found to be slightly greater when the final dose distribution is calculated with the Boltzmann solver and the median doses to the PTV and GTV are lower. This indicates that an improvement in the final dose distribution might be obtained by using the Boltzmann solver throughout the optimisation.

**Figure 11.**
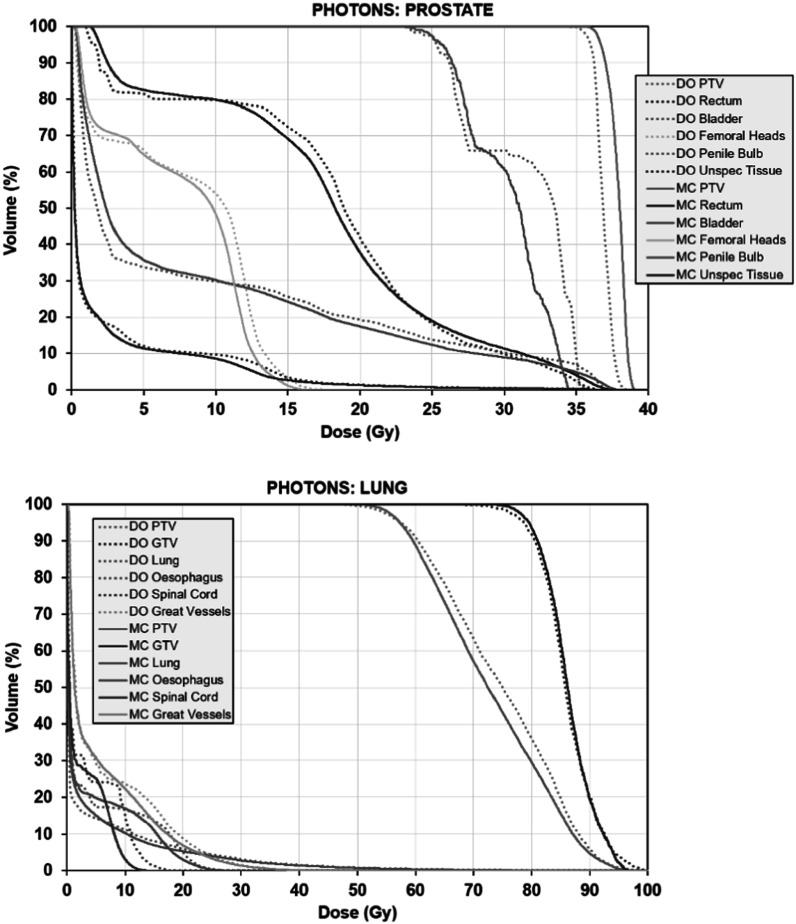
Cumulative dose–volume histograms for (a) a prostate photon treatment plan, and (b) a lung photon treatment plan, calculated using discrete ordinates (DO, dotted lines) and Monte Carlo simulation (MC, solid lines).

The comparison of LBTE with Monte Carlo simulation is shown in figure 11. The results shown are for equal monitor units. The difference in PTV dose between the two algorithms for the prostate case is approximately 3% and the difference in organ at risk dose is similar in magnitude. (The difference in dose to penile bulb is due to the field edge in relation to the small organ volume rather than due to differences in particle transport.) For the lung case, both LBTE and Monte Carlo simulation are in agreement to within around 3%. The dose to spinal cord differs by around 3 Gy.

## 4. Discussion

This study shows the possibility of implementing a discrete ordinates Boltzmann solver that is compatible with dose calculation algorithms used in the context of inverse planning. The LBTE solver is shown to be sufficiently accurate and fast for this application, thereby facilitating the eventual goal of using LBTE within the optimiser itself, so as to provide optimum inverse planning solutions.

The source models for both photons and protons are comparable to those described in the literature (Fix *et al* 2001, Aboulbanine and El Khayati 2018). It should be emphasised that the accuracy of every advanced dose calculation algorithm depends on an accurate representation of the particle fluence from the accelerator. For the case of photons, an evaluation of the source model used in this work has been carried out previously (Bedford *et al* 2022). With regard to the LBTE solver itself, there are some limitations as it is currently implemented. In particular, for photon calculations, the electron transport is treated as consisting of discrete Møller and Mott scattering cross sections, whereas a more accurate treatment can be obtained using multiple scattering theory (Goudsmit and Saunderson 1940a, 1940b, Kadri *et al* 2009), corresponding to condensed history methods in Monte Carlo simulation. There is therefore scope for further work in this area.

Estimating the accuracy of the cross sections used in the model is not straightforward. However, ICRU report 46 (International Commission on Radiation Units and Measurements 1992) tabulates quantities which use these formulae, with stated uncertainty of 1% for photon mass attenuation coefficients, 1%–2% for electron collision stopping powers and 1%–2% for proton mass stopping powers, at the energies relevant to this work. ICRU report 37 (International Commission on Radiation Units and Measurements 1984) also quotes an uncertainty of 1%–2% for electron collision stopping powers derived from the same formulae as used in this study, and ICRU report 49 (International Commission on Radiation Units and Measurements 1993) gives the uncertainty in proton mass stopping power as 1%–2% for elements and 1%–4% for compounds. Thus, the cross sections used in this study are considered to be accurate to within around 2%.

In connection with the electron cross sections, the inclusion of the shielding effect produced by the outer orbital atomic electrons could be included in the cross section, thereby removing the singularity at the forward direction. However, there are two reasons for not pursuing this in the work so far. The first is that multiple scattering theory is likely to have a greater impact on the accuracy than the shielding effect. Secondly, it is still necessary in the context of inverse planning to separate out forward scattering so as to avoid having to use a highly structured, and therefore computationally demanding, angular quadrature. Likewise, it is possible that the continuous slowing down approximation used for the proton transport can be more accurately handled by including it into the Boltzmann solver itself as a series of interactions. This is beyond the scope of the present work, although an interesting possibility for further work. Direct comparisons with Monte Carlo simulation are limited in this study because there are already many comparisons in the literature (Han *et al* 2011, Hoffmann *et al* 2018, Bedford 2019).

Another limitation of the LBTE solver is that photoelectric and pair production interactions are currently not implemented. For water-equivalent materials in the 1–10 MeV energy range used in this study, these interactions are negligible. Below this energy range, photo-electric effect increases significantly, and at higher energies, pair production increases significantly. Both of these types of interaction increase strongly with atomic number, so it is important to consider them when calculating dose in and around metals such as prostheses. In the present implementation of the Boltzmann solver, it is necessary to avoid beam entry through prostheses.

The dose calculation time is currently in the order of several minutes for a typical patient dataset with calculation on a dose grid of resolution 2.5 mm × 2.5 mm × 2.5 mm, when run on the current SPARC architecture. However, this architecture uses a hyperthreaded approach in which there are 128 threads of execution, but only 8 floating point units. This does not equate to 128 CPU cores, but depends somewhat on what tasks are required at each time instant, in practice achieving about the same speed as an 8-core Intel processor. It is expected that an order of magnitude improvement in speed can be obtained using the latest CPUs, and as the current code is written in multithreaded Java, transfer to an alternative platform is trivial. Implementation of the Boltzmann solver on a Graphics Processing Unit (GPU) is also a possibility, which should increase the performance considerably. The main limitations are likely to be that the transport sweeps are inherently sequential, with higher energy particles considered before lower energy particles, and upstream particles before downstream particles, so the parallelisation should be applied within each sweep rather than over sweeps.

A limited means of introducing robustness into the inverse planning process has been included by using the simple motion convolution model in the calculation. This is beneficial for both photons and protons, but particularly so for the latter, where range uncertainty can produce significant dose artefacts (Seco *et al* 2012). It is recognised that position uncertainty can be handled more comprehensively, but it is thought that the simple approach is sufficient for the present context, where inclusion of the LBTE into the inverse planning process is the focus. The dose distributions shown in the results section include the impact of the uncertainty modelling, thereby indicating the practical reality of the treatment plan.

The differences in the dose distribution resulting from the use of the Boltzmann solver for the final dose calculation in inverse planning indicate that some benefit might be obtained by using the LBTE solver throughout the inverse planning process rather than purely at the end. In this way, the final dose distribution should reflect the more accurate dose distribution that is used throughout the process. Work is in progress to develop this improvement in accuracy.

## 5. Conclusions

A discrete ordinates Boltzmann solver is sufficiently accurate and fast to apply to inverse planning for both photons and protons. The simple algorithm used in this study separates particle streaming from the remaining transport, thereby facilitating the use of a relatively simple angular quadrature. This approach is shown to converge reliably in several iterations of the transport sweep, and to give results which are comparable to those using a more extensive angular quadrature. Furthermore, the algorithm is consistent with a convolution algorithm for photons, or an analytical model for protons. For the lung case considered in this study, where the tumour environment is inhomogeneous, an application of the solver at the end of inverse planning shows the dose distribution to be suboptimal in comparison with the dose distribution obtained with a simple dose calculation throughout inverse planning. Application of the Boltzmann solver at intervals throughout the inverse planning is therefore worthy of investigation.

## Data Availability

The data cannot be made publicly available upon publication because they are not available in a format that is sufficiently accessible or reusable by other researchers. The data that support the findings of this study are available upon reasonable request from the authors.
